# Post-Exercise Hypotension and Its Mechanisms Differ after Morning and Evening Exercise: A Randomized Crossover Study

**DOI:** 10.1371/journal.pone.0132458

**Published:** 2015-07-17

**Authors:** Leandro C. de Brito, Rafael A. Rezende, Natan D. da Silva Junior, Tais Tinucci, Dulce E. Casarini, José Cipolla-Neto, Cláudia L. M. Forjaz

**Affiliations:** 1 Exercise Hemodynamic Laboratory, School of Physical Education and Sport, University of São Paulo, São Paulo, Brazil; 2 Neurobiology Laboratory, Institute of Biomedical Sciences, University of São Paulo, São Paulo, Brazil; 3 Department of Medicine, Division of Nephrology, School Paulista of Medicine, Federal University of São Paulo, São Paulo, Brazil; 4 Nephrology Department of Medical School, University of São Paulo, São Paulo, Brazil; 5 Post-graduate Program of Medicine, University of 9 July, São Paulo, Brazil; Oregon Health & Science University, UNITED STATES

## Abstract

Post-exercise hypotension (PEH), calculated by the difference between post and pre-exercise values, it is greater after exercise performed in the evening than the morning. However, the hypotensive effect of morning exercise may be masked by the morning circadian increase in blood pressure. This study investigated PEH and its hemodynamic and autonomic mechanisms after sessions of aerobic exercise performed in the morning and evening, controlling for responses observed after control sessions performed at the same times of day. Sixteen pre-hypertensive men underwent four sessions (random order): two conducted in the morning (7:30am) and two in the evening (5pm). At each time of day, subjects underwent an exercise (cycling, 45 min, 50%VO_2_peak) and a control (sitting rest) session. Measurements were taken pre- and post-interventions in all the sessions. The net effects of exercise were calculated for each time of day by [(post-pre exercise)-(post-pre control)] and were compared by paired t-test (P<0.05). Exercise hypotensive net effects (e.g., decreasing systolic, diastolic and mean blood pressure) occurred at both times of day, but systolic blood pressure reductions were greater after morning exercise (-7±3 vs. -3±4 mmHg, P<0.05). Exercise decreased cardiac output only in the morning (-460±771 ml/min, P<0.05), while it decreased stroke volume similarly at both times of day and increased heart rate less in the morning than in the evening (+7±5 vs. +10±5 bpm, P<0.05). Only evening exercise increased sympathovagal balance (+1.5±1.6, P<0.05) and calf blood flow responses to reactive hyperemia (+120±179 vs. -70±188 U, P<0.05). In conclusion, PEH occurs after exercise conducted at both times of day, but the systolic hypotensive effect is greater after morning exercise when circadian variations are considered. This greater effect is accompanied by a reduction of cardiac output due to a smaller increase in heart rate and cardiac sympathovagal balance.

## Introduction

Aerobic training decreases systolic/diastolic blood pressure (BP) in normotensive, pre-hypertensive and hypertensive individuals [1]. In addition, a single session of aerobic exercise produces post-exercise hypotension (PEH), i.e. a clinically relevant decrease in BP after exercise [2]. Two recent studies have shown a strong positive correlation between the magnitude of PEH and the BP decrease after aerobic training [3, 4].

Previous studies [5–8] that calculated PEH as post-pre values and compared morning and afternoon exercise, reported PEH only after exercise conducted in the afternoon, suggesting that morning exercise has no hypotensive effect. Alternatively, exercise may have the same or even a greater hypotensive effect in the morning, but that effect may have been masked by the morning circadian rise in BP. This effect can be detected if an appropriate time control condition is included in the experimental design. Thus, the first objective of this study was to compare the magnitude of PEH after exercise sessions performed in the morning and evening, controlling for circadian changes observed in control sessions (no exercise) executed at the same times of day.

The BP decrease after exercise has been attributed to either a reduction in cardiac output (CO) [9, 10] or a reduction in systemic vascular resistance (SVR) [11, 12]. These responses are influenced by changes in cardiac autonomic modulation [3, 13] and vasomotor sympathetic activity [14, 15] promoted by previous exercise. Cardiac and vasomotor sympathetic activities increase in the morning [16, 17], while vasodilatory capacity decreases [18]. These circadian patterns favor an increase in SVR that is observed throughout the morning period [19] and may blunt or offset SVR reductions after morning exercise. Thus, the second aim of this study was to investigate the hemodynamic and autonomic mechanisms of PEH after morning and evening exercise sessions, controlling for changes observed in time control sessions conducted at the same times of day.

Based on this background, the hypotheses were: 1) the net effect of exercise (the response observed after the exercise session corrected by the response observed after the control session) on PEH is greater in the morning than the evening; and 2) PEH after morning exercise is related to a reduction in CO, while after evening exercise, it is related to a decrease in SVR. As PEH is directly associated with pre-exercise BP, this study was conducted with pre-hypertensives to increase the chances for observing differences between the morning and evening periods. The results showed that morning exercise has an important and even greater hypotensive effect than evening exercise if circadian changes are considered and that mechanisms of PEH change after exercises conducted at different times of day.

## Materials and Methods

### Subjects

The inclusion criteria for the study were: males, pre-hypertensive, aged between 20 and 45 years old; nonsmokers; having no other cardiovascular or metabolic diseases; and not practicing any structured physical activity more than once a week. The exclusion criteria were: presence of obesity stage 2 or greater (in the initial protocol of the study, obesity stage 1 would also be excluded; however, this criterion was changed to stage 2 because most of the pre-hypertensive individuals have obesity stage 1); presence of any cardiovascular abnormality in resting or exercise electrocardiogram (ECG); presence of morningness or eveningness chronotype [20]; presence of poor quality of sleep [21]; and taking medications that could affect cardiovascular responses.

Subjects who fulfilled all the study criteria signed an informed written consent to participate in the study. The study was conducted according to the principles expressed in the Declaration of Helsinki and approved by the Ethics Committee of the School of Physical Education and Sport of the University of São Paulo (2011/17), and it was registered at the Brazilian Clinical Trials (www.ensaiosclinicos.gov.br-RBR-3HKQG9).

### Preliminary examination

To validate inclusion criteria and the absence of exclusion criteria, subjects underwent a set of preliminary exams. Subjects’ known health status and physical activity were assessed by their medical history and an interview about the leisure time physical activity. Body weight and height were measured (Filizola S.A, Personal, Campo Grande, Brazil) and body mass index (BMI) was calculated. Subjects were excluded if their BMI was greater than 35 kg/m^2^ [22]. Resting auscultatory BP was measured by a mercury sphygmomanometer (Uniteq, São Paulo, Brazil) three times after 5 min of seated rest in two visits, and a mean value was calculated for pre-hypertension diagnosis [23]. Subjects participated in the study only if both systolic and diastolic BP were lower than 140 and 90 mmHg, respectively; and systolic BP and/or diastolic BP were between 120 and 139 mmHg and 80 and 89 mmHg, respectively [23]. Chronotype status was assessed by the Horne and Ostberg’s questionnaire and only intermediate chronotypes (score between 45 to 65 scores) were studied [20]. Quality of sleep was assessed by the Pittsburgh Quality of Sleep Index (PQSI) and only good sleepers (scores equal to or below 5) were studied [21]. VO_2_peak was determined directly by gas analysis (Medical Graphics Corporation, CPX Ultima, Minnesota USA) during a graded maximal cardiopulmonary exercise test conducted on a cycle ergometer (Lode Medical Technology, Corival, Groningen, Netherland) with a protocol of 30 W increments every 3 min, beginning with 30 W and conducted until the subjects were unable to continue. A physician evaluated resting and exercise ECG, and subjects were excluded if they presented any abnormality.

### Experimental protocol

**Fig 1:**
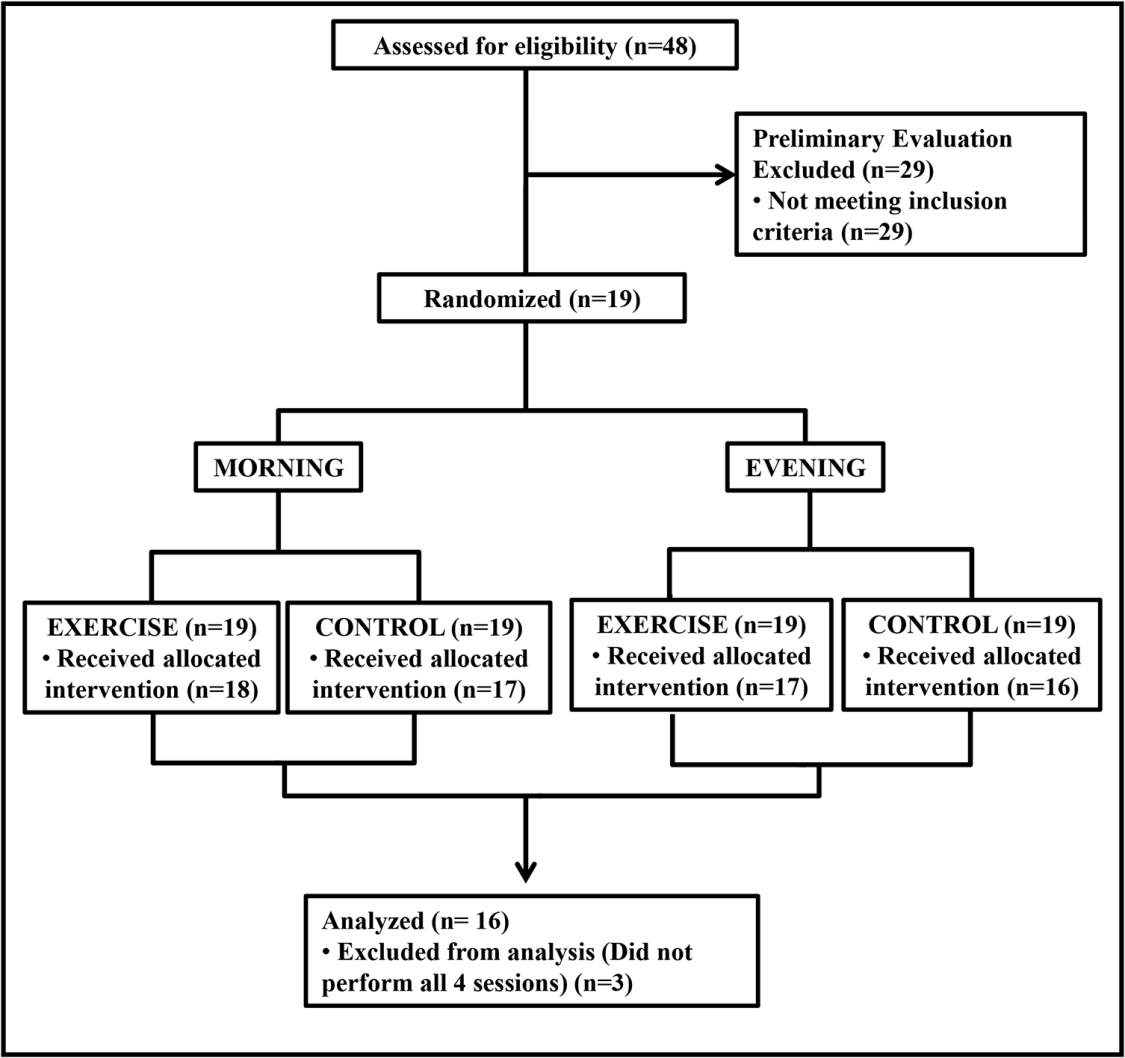
Flowchart.

**Fig 2:**
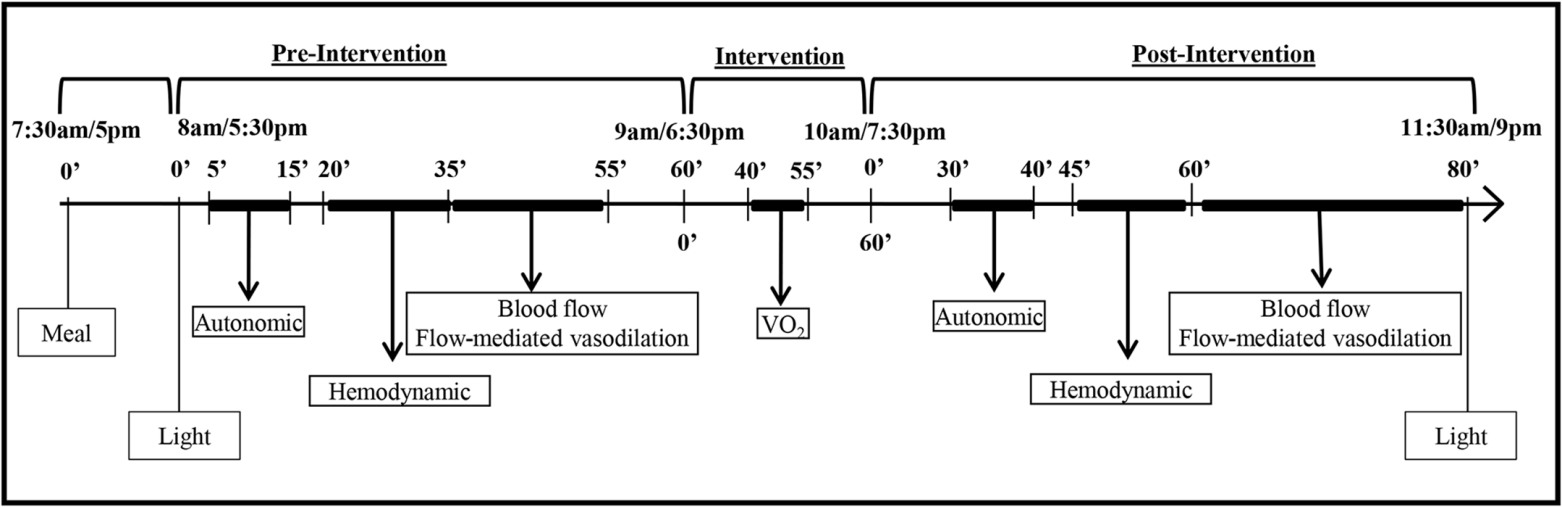
Experimental sessions.

The first participant was included in the study in February 2012 and the last in May 2013. The study followed a randomized-crossover design (Fig 1). All individuals underwent four experimental sessions conducted in a random order (simple randomization) with an interval of at least three days between them. Subjects were instructed to keep similar routines for the 24 hours before the experimental sessions. They were also instructed to avoid physical efforts and alcoholic drinks for the 24 hours before assessments. In addition, they had to arrive for the experiments having fasted for the previous 4 hours, at least. Two sessions were conducted in the morning (M– 7:30–11:30 am) and two in the evening (E– 5–9 pm). At each time of day, subjects performed one exercise (E) and one control (C) session. The protocol used in each experimental session is shown in Fig 2. In each experimental session, the subjects received a standardized meal (two cereal bars–approximately 148 Kcal and 84% carbohydrate, 8% protein and 7% fat each and 50 ml of juice–approximately 27 Kcal and 100% carbohydrate, 0% protein and 0% fat) when arriving at the laboratory. The experiments began 30 min after the ingestion of the meal.

Laboratory temperature was kept between 20 and 22°C. As this study aimed to investigate differences between morning and evening, it was essential that the subjects received the main environmental clues for each time of the day. As light is an important modulator of circadian variations, laboratory windows were kept open during experiments to allow the subjects to receive the usual amount of light at each time of the day. In addition, the ambient light level was measured at the beginning and the end of each experimental session to verify if the subjects were really exposed to the temporal light clues expected for the morning and evening moments [24].

Each session was comprised of a pre-intervention (60 min resting), an intervention (control or exercise) and a post-intervention (80 min resting) period. Autonomic and hemodynamic measurements were conducted with subjects in the sitting position. Measurements were taken at specific moments, shown in Fig 2, in the pre- and post-intervention periods. ECG and respiratory signal were registered for 10 min for autonomic assessment. Auscultatory BP, heart rate (HR) and CO were measured in triplicate (i.e. BP, HR and CO were measured in this order and this sequence of measurement was repeated three times with an interval of 3 min between the repetitions). Calf blood flow (BF) and vasodilatory response to reactive hyperemia were measured at the end of each period. During exercise, oxygen uptake (VO_2_) was measured to check exercise intensity.

#### Exercise and Control interventions

Exercise and control interventions were initiated at 9 am in the morning sessions (ME or MC) and at 6:30 pm in the evening sessions (EE or EC). Exercise consisted of pedaling on a cycle ergometer for 45 min at 50% of VO_2_peak. This protocol has been shown to promote PEH [25, 26]. In the control session, subjects rested on the cycle ergometer for the same amount of time.

### Measurements

Auscultatory systolic and diastolic BP were measured on the non-dominant arm with a mercury column (Unitec, São Paulo, Brazil). The same trained evaluator performed all BP measurements. ECG (EMG System do Brazil, EMG 030110/00B, São Paulo, Brazil) was continuously obtained and HR was registered immediately after BP assessment. CO was estimated by the indirect Fick method [27], using the CO_2_ rebreathing technique [28] and a gas analyzer (Medical Graphics Corporation CPX/Ultima, Minnesota, USA). Subjects breathed spontaneously until a steady CO_2_ production was achieved. At this moment, VCO_2_ was measured and arterial content of CO_2_ (CaCO_2_) was estimated based on the end-tidal CO_2_ pressure (PETCO_2_) measured by the gas analyzer. Afterwards, the subjects rebreathed a mixed gas with high CO_2_ concentration (8–10%) and 35% of O_2_ until an equilibrium was achieved (maximal time of 15 s). At this moment, venous CO_2_ content (CvCO_2_) was estimated based on the equilibrium of CO_2_. Then, CO was estimated by the Fick formula: CO = VCO_2_/(CvCO_2_-CaCO_2_). Stroke volume (SV) and SVR were calculated by the formulas: SV = CO/HR and SVR = Mean BP/CO. Hemodynamic data (HR, BP and CO) were measured in triplicate and a mean value for each moment (pre and post-intervention) was calculated for analysis.

For autonomic evaluation, R-R intervals assessed by ECG (EMG System do Brazil, EMG 030110/00B, Brazil) and respiratory signal assessed by a thoracic piezoelectric belt (UFI, Pneumotrace2, California, USA) were recorded for 10 min with a data acquisition system (WinDaq, DI-720, Akron, USA) using a sampling rate of 500 Hz/channel. Cardiovascular autonomic modulation was assessed by autoregressive spectral analysis of R-R interval variability following the Task Force recommendations [29]. The oscillatory components of the time series were modeled by the Levinson-Durbin recursion and the model order was chosen according to Akaike’s criterion [30]. Analyses were conducted by University of Milan software (Programma di Analisi Lineare, Milan, Italy). Low- (LF: 0.04–0.15 Hz) and high-frequency (HF: 0.15–0.4Hz) components were expressed in normalized units (nu). Although there is some debate about the physiological meaning of these components, especially the LF component, the present study accepted the suggestion of the Task Force for HR variability analyses [29], and other original studies [31, 32]. The LF_R-R_nu was accepted as a marker of predominant cardiac sympathetic modulation, TV_R-R_ and HF_R-R_nu as markers of predominant cardiac parasympathetic modulation and LF/HF ratio as representative of cardiac sympathovagal balance.

Calf BF was assessed by venous occlusion plethysmography (Hokanson, AI6, Bellevue, USA), as previously described [33]. Cuffs were placed on the ankle and the thigh and a mercury-in-silastic strain gauge was placed at the biggest portion of the calf. The subject’s leg was elevated and placed on a platform at hip height. During measurements, the ankle cuff was inflated to 200 mmHg, while the thigh cuff was inflated to 60 mmHg for 10 s every 20 s. BF was measured for 4 min and a mean value was calculated. BF collected under the same conditions and with the same technique showed good reliability (1.89±1.17 vs. 1.81±1.00, p>0.05 and intra-class correlation coefficient 0.791, p = 0.001) (non-published data). The calf vascular resistance (CVR) was determined as the ratio between mean BP and calf BF.

The vasodilatory response to reactive hyperemia was also assessed [34]. After BF measurement, BF to the calf was totally occluded for 5 min by inflating the thigh cuff to 200 mmHg ensuring a complete occlusion [35, 36]. Then, this cuff was released and calf BF was measured for 4 min as described above. The vasodilatory response was assessed by the area under the curve (calf AUC) calculated from BF measurements taken after hyperemia.

### Statistical analysis

Considering an α error of 0.05 and a power of 90%, the minimum sample size needed to detect a difference of 4 mmHg in BP, considering a SD = 3 mmHg, is 10 subjects, and to detect a difference of 0.32 l/min in CO, considering a SD = 0.32 l/min, is 11 subjects [13].

Data normality was tested by Shapiro-Wilk test (SPSS, Illinois, USA). Logarithm transformation was applied when needed to achieve normality (for TV_R-R_ and LF/HF ratio).

At each time of day (morning and evening), exercise effect was assessed by a two-way ANOVA for repeated measures (Statsoft, Statistic for windows, USA), considering sessions (C and E) and stages (pre- and post-intervention) as the main factors.

The net effect of exercise for each time of day was calculated by: [(post-pre E)—(post-pre C)]. Student t-test was used to compare the net effects of exercise in the morning and evening.

Post-hoc analyses were conducted by Newman Keuls’ test whenever necessary. A p≤0.05 was set as significant for all analyses. Data are presented as mean±standard deviation.

## Results

The flowchart is shown in Fig 1. Seventy-nine subjects volunteered for the study, but 31 did not fulfill the criteria. Thus, 48 subjects signed the written consent, but 29 subjects were excluded in the preliminary exams (25 for not confirming pre-hypertension and 4 for poor sleep quality). Therefore, 19 subjects initiated the experiments, but 3 dropped out due to personal reasons. The final sample was composed by 16 subjects whose characteristics are presented in Table 1.

**Table 1:** Physical and functional characteristics of the sample.

N	16
Age (yrs)	32±7
Height (m)	1.74±0.07
Weight (kg)	88.2±12.8
Body mass index (kg m^-2^)	28.9±2.8
Resting systolic BP (mmHg)	124±6
Resting diastolic BP (mmHg)	84±4
Resting mean BP (mmHg)	97±4
Heart rate (bpm)	73±8
Chronotype	54.8±7.7
Sleep quality	3.8±1.1
Maximal Workload (watts)	191±33
VO_2_ peak (ml.kg^-1^.min^-1^)	30.9±6.2
Maximal heart rate (bpm)	174±14
Maximal systolic BP (mmHg)	197±16

Values in mean±SD. BP—blood pressure. VO_2_ –oxygen uptake

Four subjects began the study in each one of the experimental sessions (MC, ME, EC, EE). Laboratory ambient light increased during the morning (460±51 vs. 821±197 lux, p<0.05) and decreased during the evening (702±239 vs. 395±84 lux, p<0.05) sessions. During exercise, workload was 80±30 watts, and VO_2_ was similar in ME and EE sessions (1338±202 and 1321±189 ml/min, respectively, p>0.05), corresponding to similar exercise intensities (50.6±0.1 and 50.0±0.1% of VO_2_peak, respectively, p>0.05).

### Pre-intervention values

Pre-intervention values were similar among the four experimental sessions for all variables, except HR and calf AUC. Pre-intervention HR and calf AUC were greater in the evening than in the morning (MC = 69±7 and ME = 67±6 vs. EC = 71±6 and EE = 71±7 bpm, p = 0.01; and MC = 260±188 and ME = 379±260 vs. EC = 421±193 and EE = 363±212 U, p = 0.03).

### Exercise effect at each time of day

In the morning sessions (Table 2), systolic BP increased significantly after MC and decreased after ME, while diastolic BP and mean BP increased significantly after MC and did not change after ME. CO decreased while SVR increased after both sessions; however, CO responses were greater after ME than MC. SV decreased after both sessions, and the decrease was greater after ME. HR did not change after MC and increased after ME. TV_R-R_ increased after MC and did not change after ME, while HF_R-R_nu, LF_R-R_nu and LF/HF did not change after either session. CVR increased after MC and did not change after ME, while calf AUC increased similarly after both sessions.

**Table 2:** Hemodynamic, autonomic and vascular data assessed pre and post interventions in the morning control (MC) and exercise (ME).

		PRE	POST
Systolic BP (mmHg)	MC	121±10	124±9*
	ME	122±9	118±3* #
Diastolic BP (mmHg)	MC	84±7	87±6*
	ME	84±8	84±8#
Mean BP (mmHg)	MC	96±7	100±7*
	ME	97±7	96±8#
Cardiac output (l/min)	MC	4.92±0.59	4.42±0.55*
	ME	4.96±0.41	4.01±0.78* #
Systemic vascular resistance (U)	MC	20±3	23±3*
	ME	20±2	25±5*
Stroke volume (ml)	MC	74±11	67±13*
	ME	75±10	55±10* #
Heart rate (bpm)	MC	69±7	67±8
	ME	67±6	73±7* #
Total variance _R-R_ (ms^2^)	MC	7.6±0.9	8.3±0.9*
	ME	7.8±0.9	7.7±1.0#
LF_R-R, (_nu)	MC	67±16	66±16
	ME	67±18	69±18
HF_R-R, (_nu)	MC	29±15	29±15
	ME	26±16	26±17
lnLF/HF	MC	1.01±0.98	0.96±0.89
	ME	1.07±1.02	1.21±1.11
CVR (U)	MC	90±31	111±35*
	ME	101±38	97±33#
AUC (U)	MC	260±188	365±145*
	ME	379±260	414±210*

Values in mean±SD. BP—blood pressure. LF—low frequency. HF—high frequency. CVR–calf vascular resistance. AUC–area under the curve.

* significantly different from pre-intervention (P≤0.05)

# significantly different from control session (P≤0.05).

In the evening sessions (Table 3), systolic BP decreased after both sessions, and the decrease was greater after EE. Diastolic BP increased significantly after EC and did not change after EE, while mean BP decreased only after EE. CO decreased while SVR increased similarly after both sessions. SV decreased after both sessions, but the decrease was greater after EE. HR decreased after EC and increased after EE. TV_R-R_ did not change in either sessions, but it was lower after EE than EC at post-intervention measurement. HF_R-R_nu increased while LF_R-R_nu and LF/HF decreased after EC, while they did not change after EE. CVR increased after EC and did not change in EE, while calf AUC increased only after EE.

**Table 3:** Hemodynamic, autonomic and vascular data assessed pre and post-interventions in the evening control (EC) and exercise (EE).

		PRE	POST
Systolic BP (mmHg)	EC	121±9	119±9*
	EE	123±9	117±4* #
Diastolic BP (mmHg)	EC	84±7	86±7*
	EE	84±6	83±8#
Mean BP (mmHg)	EC	96±7	97±7
	EE	97±6	95±6* #
Cardiac output (l/min)	EC	4.90±0.75	4.15±0.53*
	EE	5.04±0.63	4.44±0.65*
Systemic vascular resistance (U)	EC	20±3	24±4*
	EE	19±3	22±4*
Stroke volume (ml)	EC	70±12	65±12*
	EE	73±11	59±8* #
Heart rate (bpm)	EC	71±6	65±6*
	EE	71±7	75±6* #
Total variance _R-R_ (ms^2^)	EC	7.5±0.7	7.7±0.7
	EE	7.6±0.9	7.3±1.1#
LF_R-R, (_nu)	EC	74±15	58±15*
	EE	68±21	73±19#
HF_R-R, (_nu)	EC	21±13	37±15*
	EE	28±18	20±18#
lnLF/HF	EC	1.41±0.92	0.52±0.75*
	EE	1.11±1.12	1.67±1.30#
CVR (U)	EC	85±24	110±42*
	EE	89±22	86±24#
AUC (U)	EC	421±193	443±270
	EE	363±212	506±310*

Values in mean±SD. BP—blood pressure. LF—low frequency. HF—high frequency. CVR–calf vascular resistance. AUC–area under the curve.

* significantly different from pre-intervention (P≤0.05)

# significantly different from control session (P≤0.05).

### Net effects of exercise in the morning and evening

**Fig 3:**
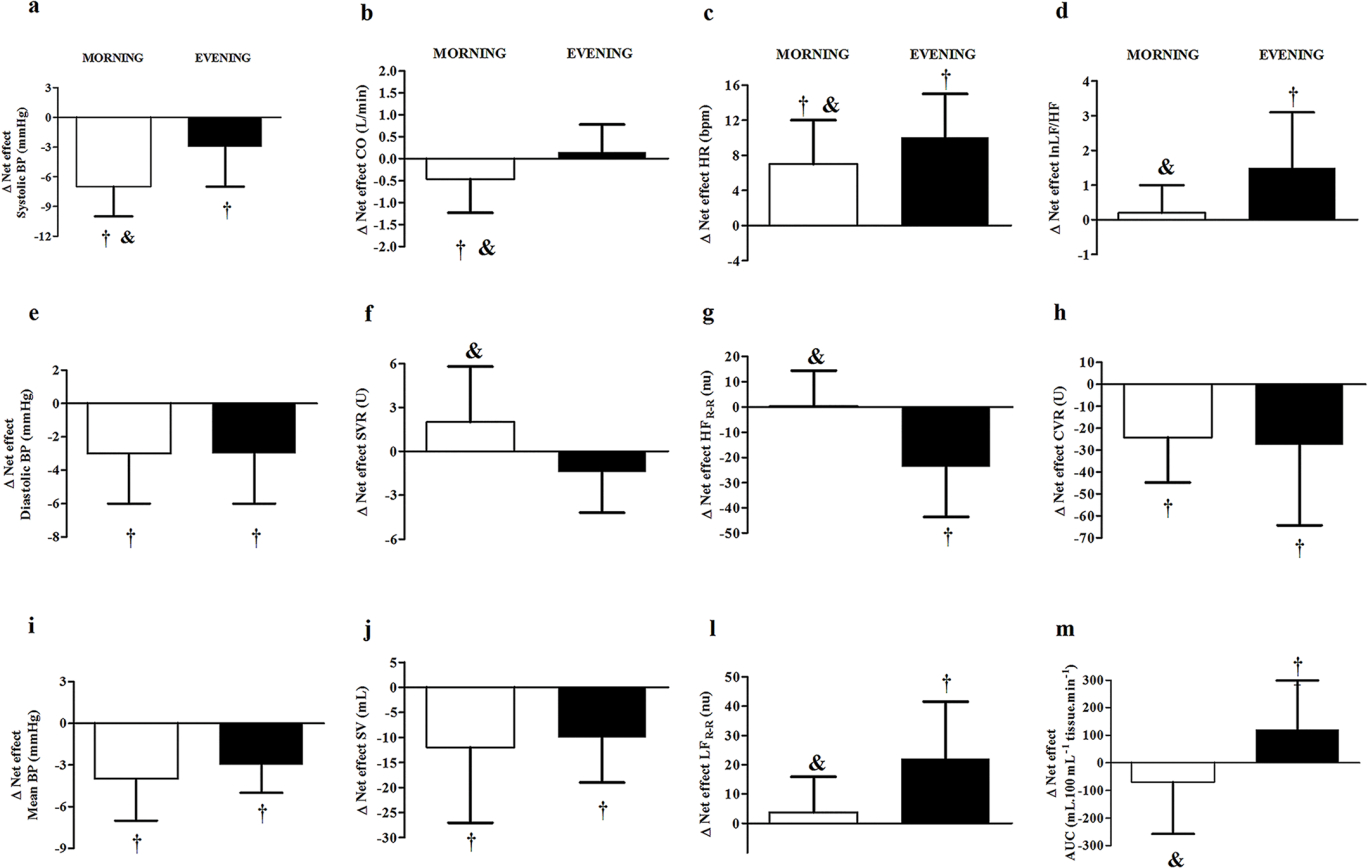
Comparison between the net effect of exercise in the morning and evening. (a) Systolic blood pressure (SBP), (b) cardiac output (CO), (c) heart rate (HR), (d) logarithmic of low to high frequency ratio of R-R interval variability (lnLF/HF), (e) diastolic blood pressure (DBP), (f) systemic vascular resistance (SVR), (g) normalized high-frequency component of R-R interval variability (HF_R-R_), (h) calf vascular resistance (CVR), (i) mean blood pressure (MBP), (j) stroke volume (SV), (l) normalized low-frequency component of R-R interval variability (LF_R-R_) and (m) calf vascular resistance (CVR) and area under the curve of calf blood flow response to reactive hyperemia (calf AUC). , , , , , , † Significant net effect (P≤0.05), & significantly different from evening net effect (P≤0.05).

Morning and evening net effects of the exercise are shown and compared in Fig 3. The net effect of exercise reducing systolic BP was observed at both times of day and was significantly greater in the morning (-7±3 vs. -3±4 mmHg, p<0.05) (Fig 3A). The net effects of the exercise reducing diastolic and mean BP were similar at both times of day (-3±3 vs. -3±3 mmHg, and -4±3 vs. -3±3 mmHg) (Fig 3A and 3I). The net effect of exercise reducing CO occurred only in the morning (-0.460±0.771 vs. +0.148±0.633 l/min, p<0.05) (Fig 3B), while the net effect of exercise increasing SVR was not significant at either time of the day but differed between morning and evening exercises (+2.0±3.8 vs. -1.4±2.8 U, respectively, p<0.05) (Fig 3F). The net effect of exercise reducing SV was similar at both times of day (-12±15 vs. -10±9 mL, p>0.05) (Fig 3J), while the net effect of exercise increasing HR occurred at both times of day, but was lower in the morning (+7±5 vs. +10±5 bpm, p<0.05) (Fig 3C). The net effect of exercise decreasing TV_R-R_ was similar at both times of day (-0.8±0.6 vs. -0.6±0.6 ms^2^), while the net effects of the exercise decreasing HF_R-R_nu and increasing LF_R-R_nu and LF/HF were observed only in the evening (0±14 vs. -24±20 nu; +3±12 vs. +22±19 nu, and +0.2±0.8 vs. +1.5±1.6, p<0.05, respectively) (Fig 3G, 3l and 3D). The net effect of the exercise decreasing CVR was similar in the morning and evening (-24.2±20.5 vs. -27.7±36.6 U, p<0.05) (Fig 3H), while the net effect of exercise increasing calf AUC occurred only in the evening (-70±188 vs. +120±179 U, p<0.05) (Fig 3M).

## Discussion

The main finding of this study is that, although aerobic exercise conducted both in the morning and evening produced PEH, the hypotensive effect of exercise was greater for systolic BP after morning exercise when circadian variations of BP were taken into account. In addition, this greater morning systolic PEH occurred due to a decrease in CO secondary to a blunted increase in HR, which was mediated by a lower sympathovagal balance increase after exercising at this time of day. In contrast, in the evening, PEH was accompanied by a net effect of exercise increasing calf vasodilatory response.

The novelty of the current study was to show that morning exercise produced greater systolic PEH than the same exercise performed in the evening when circadian variation of BP is taken into account. Although the main difference may not seem large (-7±3 vs. -3±4 mmHg, p<0.05), it is important to mention that after morning exercise almost all of the subjects (14) had a net decrease in systolic BP greater than 4 mmHg while only 8 subjects (half of the sample) showed this amount of decrease after evening exercise. Previous studies [6–8] that calculated PEH as post-pre exercise values, concluded that morning exercise was less effective in decreasing BP during recovery. In the present study, if PEH was calculated as post-pre difference, PEH would also be lower after the morning than the evening exercise (-3.6±3.0 and -5.1±3.3 mmHg, respectively, p<0.05). However, when the time control condition is considered, the results are the opposite, with a greater hypotensive effect after morning exercise. Thus, the results of this study clearly show that interpretation of the hypotensive effects of previous exercise may be completely different if the superimposed circadian pattern is not considered in the study design. By including the control sessions, this study was able to show that although morning exercise produces a lower post-pre decrease in systolic BP than evening exercise, it abolished the morning systolic BP increase, which implies a significantly greater hypotensive effect when exercise is performed in the morning.

Regarding diastolic and mean BP, the net effects of exercise were similar after morning and evening exercises. In addition, differences between post-pre exercise values were also similar for diastolic and mean BP in the morning and evening (0±3 vs -1±3 mmHg, p>0.05; and -1±2 vs -2±3 mmHg, p>0.05, respectively). Actually, after the control sessions at both times of day, diastolic BP increased similarly, which may be explained by the increase in SVR observed at both times of day. Previous studies have already reported increases in SVR and diastolic BP throughout control sessions conducted in the sitting position in the morning [25], the evening [13] and both [37]. The sitting position imposes an orthostatic stress that decreases venous return and deactivates the cardiopulmonary reflex, which increases peripheral sympathetic activity, SVR and diastolic BP [38]. In the evening, these effects may have overcame the circadian variation of BP, explaining the absence of difference in diastolic BP behavior after morning and evening control sessions. Exercise executed at both times of day was able to abolish the orthostatic effect of the sitting position on diastolic BP.

The greater systolic BP reduction after morning exercise was accompanied by a net reduction in CO that occurred only in the morning, and was not overcome by an increase in SVR. These results are in accordance with studies that observed no decrease in SVR after morning exercise [7, 8, 10]. The CO decrease after morning exercise was due to a reduction in SV that has already been reported [9, 10, 39, 40] and has been attributed to a reduction in pre-load [2]. The greater net decrease in CO after morning exercise was mainly due to the net increase in HR being lower after morning than after evening exercise, which did not compensate for the SV decrease. HR did not change throughout the morning control condition, which may be explained by baroreflex responses to diastolic BP increases [38], counterbalancing the morning circadian increase in HR. In contrast, HR decreased throughout the evening control session, which may be due to the combination of the baroreflex responses to diastolic BP increases, superimposed on the circadian alterations of HR in the evening [41]. After exercise at both times of day, HR increased, which has been consistently reported [14, 25, 40]. Despite the slightly greater increase observed after morning exercise in relation to pre-exercise values, the net effect of exercise increasing HR was lower in the morning due to the notable differences between morning and evening control conditions.

HR is mainly regulated by the cardiac autonomic system. To our knowledge, this is the first study to investigate whether time of day influences cardiovascular autonomic responses after exercise. In the current study, no significant change in cardiac autonomic modulation was observed after morning sessions, except for TV_R-R_ that increased in MC and did not change in ME. Thus, morning exercise blunted TV_R-R_ increase, which may be related to the net effect of exercise increasing HR at this time of day. In contrast, in the evening, after EC, markers of cardiac parasympathetic modulation (TV_R-R_ and HF_R-R_) increased while markers of cardiac predominant sympathetic modulation (LF_R-R_) decreased, decreasing sympathovagal balance (LF/HF), which is in accordance with the decrease in HR. In addition, after the EE, these markers did not change, suggesting that the net effect of exercise conducted in the evening was an increase in sympathetic and a decrease in vagal cardiac modulations, which explains the greater net increase in HR observed after exercise conducted in the evening.

Although CVR was similarly reduced by previous exercise at both times of day, only evening exercise increased the calf vasodilatory response to reactive hyperemia, suggesting that exercise had greater vascular effects when performed in the evening. It is known that vasodilatory responses to reactive hyperemia are greater in the evening than in the morning [18], and this circadian behavior may have facilitated the vascular effects of exercise at this time of day. Additionally, some studies reported that local vasodilation responses to exercise are only observed in the afternoon [5, 8], which may be due to the lower presence of constrictor substances, such as angiotensin II, at this time of day [42]. Since baroreflex sensibility is greater in the evening than in the morning [41], the greater post-evening exercise vasodilatory response could have resulted in a greater tendency to decrease BP, leading to a greater baroreflex-mediated increase in cardiac sympathetic modulation after the evening exercise, which might explain the greater increase in HR observed after the evening than the morning exercise. Concerning the differences in mechanisms contributing to morning vs. evening PEH, it is possible that the greater vasodilatory responsiveness induced by evening exercise produced a greater baroreflex mediated increase in sympathetic activation.

The present study has some limitations. Firstly, only sedentary men were investigated. As gender and training status have been shown to influence PEH mechanisms [43], results should not be directly extrapolated to women and physically active subjects. The sample included overweight and stage 1 obese pre-hypertensive men. It is known that the presence of weight excess can alter cardiovascular responses to exercise [44]; however, weight excess is present in approximately 65% of pre-hypertensive subjects [45]. Results might also be different if exercises were conducted earlier or later in the morning and evening since circadian hemodynamic variation occurs throughout the day [19, 46]. Other exercise modes might also promote different responses since PEH seem to be different after aerobic and resistance exercise [13, 47]. Regarding aerobic exercise protocols, although intensity and duration might influence the magnitude of post-exercise responses [25, 48], they do not seem to change the mechanisms involved in these responses. Thus, it is not probable that the influence of time of day on PEH would be changed by aerobic exercise with different durations or intensities. However, future studies should specifically investigate other exercise modes and protocols. Although conducted with pre-hypertensive subjects, it is not expected that there would be different responses at different times of day among individuals of differing BP levels, except for non-*dipper* patients who have a disruption of circadian BP rhythms. Nevertheless, results could differ in subjects receiving medications that affect hemodynamic and autonomic parameters. Regarding measurements, the calf cuff was inflated to 200 mmHg for reactive hyperemia as this pressure assured a complete occlusion in pre-hypertensives. The same pressure was kept in all the sessions and, thus, this value should not have affect the results [36]. Finally, an interesting aspect of current data is that there were few differences in pre-intervention measures done in the morning and evening other than HR and calf vasodilatory response to reactive hyperemia. The absence of other differences may be attributed to the specific times chosen for the evaluations, in conjunction with the fact that subjects had traveled from their homes to the laboratory before exercise. Thus, morning evaluations were performed when subjects had already been awake and active for more than one hour. Nevertheless, in clinical practice, when subjects exercise in the morning, they usually have to wake-up and go to exercise facilities, which may take some time. Thus, the experimental protocol mimics real life exercise, increasing the applicability of the results.

As a clinical perspective, a greater number of cardiac events occur in the morning than at other times of day, and there are some concerns about doing exercise at this time of day [49]. In opposition to this view, the present results suggest that morning exercise may result in greater PEH, lower cardiac load and lower cardiac sympathovagal modulation after its execution (greater systolic BP decrease, lower HR increase and no change in LF/HF) than evening exercise, suggesting a lower cardiovascular risk after exercise when it is performed in the morning. In addition, for subjects who present high BP morning surges, the present results suggest that morning BP increase might be blunted by previous exercise. However, the reproducibility of these results in populations with high cardiovascular risk should be tested. Regarding the specific applicability of the results concerning the recommendation of morning or evening exercise for acutely controlling BP in pre-hypertensive subjects, some discussion should be done. Although the hypotensive effect of exercise was greater after the morning exercise, suggesting it is better to exercise in the morning, the clinical relevance of PEH also depends on hypotensive effect duration; i.e. PEH should persist for many hours after the exercise while the subjects are performing their daily activities [50, 51], which was not assessed in the present study. Thus, future investigations should evaluate ambulatory BP after morning and evening exercise for supporting the recommendation of morning or evening exercise in pre-hypertensives.

In conclusion, a single session of aerobic exercise promotes PEH regardless of the time of day at which it is performed. However, morning exercise promotes a greater reduction in systolic BP during the recovery period. This greater systolic hypotensive effect in the morning is accompanied by a reduction of CO due to a SV reduction and a lower increase in HR due to a lower increase in sympathovagal balance after morning exercise. In addition, there was a smaller vasodilatory response after morning exercise.

## Supporting Information

S1 CONSORT Checklist CONSORT checklist. (DOCX)

S1 File Trial Original Study. (DOCX)

S2 File Trial English Study. (DOCX)

S3 File English Review. (PDF)
