# Supplementary material for: Post-Exercise Hypotension and Its Mechanisms Differ after Morning and Evening Exercise: A Randomized Crossover Study
Source: PLoS One. 2015 Jul 17;10(7):e0132458. doi: 10.1371/journal.pone.0132458 (PMC4506120; doi:10.1371/journal.pone.0132458)
Supplement: S1 File — (DOCX) [file pone.0132458.s002.docx]

# PROJETO DE PESQUISA

*(no máximo 20 páginas)*

#### 1. Informações Gerais

**Título:** Hipotensão Pós-Exercício Aeróbico e Seus Mecanismos Hemodinâmicos e Neurais em Pré-Hipertensos: influência do horário do dia e associação com a regulação endócrina circadiana.

**Pesquisador responsável:** Profa. Dra. Cláudia Lúcia de Moraes Forjaz

**Pesquisador gerente:** Leandro Campos de Brito

**Departamento envolvido no estudo:** Biodinâmica do Movimento Humano

**2. Introdução**

| A prática regular de exercícios físicos, principalmente aeróbicos, tem sido adotada como terapia não medicamentosa para a prevenção e o combate da hipertensão arterial, sendo recomendada por diretrizes nacionais e internacionais tanto para indivíduos hipertensos quanto para sujeitos propensos a desenvolver essa doença, como por exemplo, sujeitos pré-hipertensos (12, 59). Os efeitos hipotensores do exercício aeróbico são observados após um período de treinamento (10, 19), mas também têm sido relatados em resposta a apenas uma sessão de exercício (11, 21, 23, 24, 27, 47, 57). De fato, após o término de uma sessão de exercício aeróbico, os níveis de PA diminuem e permanecem inferiores aos observados no período que antecede o exercício ou ainda àqueles avaliados em um dia controle, em que não houve a realização do exercício. Este fenômeno tem sido denominado na literatura de hipotensão pós-exercício (38).  Ao longo dos anos, a hipotensão pós-exercício vem sendo estudada por vários grupos, incluindo o nosso e, em 2004, o Colégio Americano de Medicina do Esporte afirmou sua relevância clínica para a hipertensão por apresentar magnitude significante e duração prolongada (57). Nesse contexto, um estudo do nosso laboratório encontrou, em normotensos, redução dos valores da PA por 24 horas pós-exercício (24). Resultados similares foram observados por Rondon et al. (2002) em hipertensos. Com relação à magnitude da hipotensão pós-exercício, em revisão sobre o tema MacDonald (2002) reportou quedas na PA sistólica/diastólica de 8/9, 14/9 e 10/7 mmHg para normotensos, limítrofes e hipertensos, respectivamente.  Apesar de ter comprovada relevância clínica, tanto a duração quanto a magnitude da hipotensão pós-exercício variam muito entre os estudos, o que sugere que diversos fatores possam afetá-las. Nesse sentido, o presente estudo investigará o possível efeito do ritmo circadiano sobre a hipotensão pós-exercício, mais especificamente do período do dia no qual o exercício é executado.  Estudos prévios do grupo da Dra. Jones (32, 34-36) sugerem que o exercício aeróbico realizado pela manhã promove menor redução da PA ou mesmo não causa esse efeito, enquanto que o exercício realizado no fim da tarde oferece maior queda. Porém, esses estudos foram desenvolvidos em normotensos. Além disso, não houve uma sessão controle que permitisse avaliar as alterações impostas à PA pelo decorrer do tempo, o que pode ter limitado a interpretação correta dos resultados, visto que no período matutino os níveis de PA costumam se elevar (16, 49) de modo que a ausência de elevação da PA após exercício matutino pode, na realidade, representar um efeito hipotensor tão expressivo quanto à redução observada pós-exercício no período da tarde. Dessa forma, adotar uma situação controle é de suma importância nesse contexto. Assim, avaliar a influência do horário do dia nas respostas da PA pós-exercício perante a comparação com uma sessão controle será o primeiro objetivo do presente estudo.  Os determinantes hemodinâmicos da hipotensão pós-exercício aeróbico também têm sido estudados, contudo, os resultados obtidos até o momento são inconclusivos. Alguns estudos encontraram redução dos valores de PA pela diminuição do débito cardíaco (5, 18, 26, 30, 45, 58, 62), enquanto que outros observaram queda da resistência vascular periférica como o principal determinante desse fenômeno (13, 20, 27-29, 31, 33, 40, 42, 43, 47, 48, 61, 64). Novamente, vários fatores podem ser responsáveis por essa diferença nas respostas, sendo que o estudo corrente investigará o possível efeito do período do dia.  Quando a queda da PA pós-exercício está associada à redução do débito cardíaco, essa normalmente é atribuída à redução do volume sistólico em decorrência de uma menor pré-carga, sendo que essa diminuição não é compensada apesar do aumento da freqüência cardíaca desencadeada pela permanência da atividade nervosa simpática elevada para o coração durante o período de recuperação (18, 26, 30, 45, 58, 62). É interessante observar que apesar da redução da pré-carga desativar o reflexo cardiopulmonar, o que deve resultar em aumento da resistência vascular periférica (3), esse aumento não ocorre após o exercício aeróbico, o que sugere que mesmo quando a diminuição da PA pós-exercício se deve a redução do débito cardíaco, o exercício também tem efeito sobre a periferia, atenuando a elevação da resistência vascular periférica.  Por outro lado, quando a redução da resistência vascular periférica é responsável pela hipotensão pós-exercício, essa redução é atribuída à manutenção da vasodilatação induzida pelo exercício durante o período de recuperação, o que pode ocorrer em função da redução da atividade simpática para a periferia (7, 22) bem como pela secreção de substâncias vasodilatadoras (43, 48, 52), ou ainda pela redução da responsividade vascular a estímulos vasoconstritores (28).  Considerando-se os diferentes períodos do dia, sabe-se que as variáveis hemodinâmicas variam ao longo das 24 horas. Assim, no período da manhã, quando o indivíduo acorda a PA se eleva. Esse aumento reflete a ativação de alguns mecanismos pressores, como a elevação da atividade nervosa simpática, que no coração aumenta a freqüência cardíaca (25, 44) e na periferia aumenta a resistência vascular periférica (55). Além disso, nesse horário, a sensibilidade barorreflexa está reduzida (65, 67), o que facilita o aumento da PA. Do mesmo modo, a função endotelial está prejudicada no período matutino (37, 53), dificultando a vasodilatação e colaborando para a elevação da resistência vascular periférica e da PA. Dessa forma, como diversas variáveis neurais, hormonais e hemodinâmicas apresentam comportamento diferente ao longo do dia, é possível que esses parâmetros respondam de forma diferente após o exercício realizado de manhã e ao final da tarde.  Nos estudos realizados pelo grupo da Dra Jones, a menor hipotensão pós-exercício observada no período da manhã foi atribuída a uma maior resistência vascular periférica (32, 35). Entretanto, esses autores não investigaram simultaneamente outros parâmetros hemodinâmicos, e não avaliaram a regulação neural e hormonal da hipotensão pós-exercício. Dessa forma, a avaliação da influência dos horários do dia nos mecanismos hemodinâmicos e neurais na hipotensão pós-exercício será outro objetivo desse estudo.  As alterações circadianas observadas nas diferentes funções fisiológicas estão sob o controle de um relógio biológico que, no ser humano, é regido principalmente pelo núcleo supraquiasmático. Nesse núcleo, observa-se a presença de populações de neurônios pré-simpáticos e pré-parassimpáticos (50), o que o torna responsável pela integração do relógio circadiano central com a regulação autonômica cardiovascular central e periférica (60). Além disso, o principal marcador endógeno do ciclo dia e noite em interação com o núcleo supraquiasmático é a melatonina. Este hormônio, produzido na glândula pineal, pode atingir níveis noturnos 30 vezes maiores do que o matinal (6). Além de marcar o ciclo dia e noite, esse hormônio tem ações sobre o controle barorreflexo, diminuindo seu set-point (39) e sobre os vasos, relaxando-os (4, 56). Através das ações da melatonina, o núcleo supraquiasmático influencia os determinantes hemodinâmicos da PA (60).  Não somente a melatonina, mas também outros hormônios responsáveis pela regulação da PA apresentam um ritmo circadiano. É o caso da norepinefrina e da angiotensina II, que estão elevadas pela manhã (15, 17), enquanto que os níveis de vasopressina encontram-se reduzidos pela manhã (2), o que explica o comportamento do volume sistólico nesse horário do dia (41, 69). Dessa forma, torna-se interessante observar a relação entre os marcadores hormonais do ritmo circadiano e as respostas fisiológicas pós-exercício em diferentes períodos do dia. Este seria então, o outro objetivo do presente estudo.  Perante a argumentação apresentada anteriormente, a hipótese levantada para esta investigação é que no período da manhã, as respostas hipotensivas pós-exercício corrigidas pelas alterações observadas na sessão controle serão semelhantes ou um pouco menores que à tarde. Todavia, no período da manhã, essa resposta estará relacionada à redução do débito cardíaco, devido à queda do volume sistólico, visto que a diminuição da resistência vascular periférica pós-exercício estará prejudicada nesse horário. Além disso, as diferenças nas respostas observadas nos diferentes períodos avaliados se correlacionarão com os níveis plasmáticos de norepinefrina, angiotensina II, vasopressina e melatonina. |
| --- |

**3. Objetivos Gerais e Específicos**

| **3.1 Geral**  Analisar a influência do horário do dia no comportamento e nos determinantes hemodinâmicos, vasculares e neurais da hipotensão pós-exercício, relacionando essa influência às características neuro-humorais observadas em cada período do dia.    **3.2 Específicos**  Comparar, em indivíduos pré-hipertensos, as respostas observadas após uma sessão de exercício aeróbico realizado de manhã e ao final da tarde, em relação à:   1. Pressão arterial clínica e de 24 horas; 2. Determinantes hemodinâmicos: débito cardíaco, resistência vascular periférica, volume sistólico e freqüência, cardíaca; 3. Determinantes neurais: modulação simpática e parassimpática para o coração, modulação simpática vasomotora e sensibilidade barorreflexa espontânea; 4. Fluxo sanguíneo e função endotelial;   Relacionar as respostas hemodinâmicas, autonômicas e vasculares pós-exercício observadas pela manhã e no final de tarde com os níveis plasmáticos de noradrenalina, epinefrina, angiotensina II, vasopressina e melatonina medidos nesses dois períodos do dia. |
| --- |

**4. Métodos**

| **4.1 Casuística**  Participarão do estudo 16 indivíduos jovens, do sexo masculino, na faixa etária entre 20 e 40 anos, com níveis de PA alterados, não obesos e não praticantes regulares de exercícios físicos. Nenhum dos voluntários poderá fazer uso de medicamentos que afetem o sistema cardiovascular. Os voluntários serão esclarecidos sobre os procedimentos experimentais e possíveis riscos envolvidos no estudo e, então, assinarão o Termo de Consentimento Livre e Esclarecido. Este estudo será submetido ao Comitê de Ética em Pesquisa da Escola de Educação Física e Esporte da Universidade de São Paulo.  4.2 Procedimentos preliminares  4.2.1 Diagnóstico de níveis alterados de PA  A PA será aferida três vezes em cada braço, após cinco minutos de repouso com o voluntário na posição sentada, como sugere o The Seventh Report of the Joint National Committee on Prevention, Detection, Evaluation, and Treatment of High Blood Pressure: the JNC 7report, (2003). Esse procedimento será repetido em duas visitas ao laboratório, utilizando-se um esfigmomanômetro de coluna de mercúrio e considerando-se as fases I e V dos sons de Korotkoff para a identificação das PA sistólica e diastólica, respectivamente. A média dos seis valores medidos em cada braço será calculada e a média obtida no braço de maior PA será considerada para a análise. Só serão incluídos no estudo, os indivíduos com valores de PA sistólica e diastólica inferiores a 140 e 90 mmHg, respectivamente. Além disso, como o estudo será realizado com indivíduos pré-hipertensos, uma das duas pressões ou ambas deverão estar: se a PA sistólica entre 120 e 139 mmHg e se a PA diastólica entre 80 e 89 mmHg.  4.2.2 Diagnóstico de obesidade  O peso e a estatura dos voluntários serão medidos numa balança (Welmy) e o índice de massa corporal (IMC) será calculado pelo quociente entre o peso (kg) e o quadrado da estatura (m^2^). Apenas participarão do estudo os voluntários com IMC inferior a 30kg/m^2^ (51). Os indivíduos caracterizados como obesos serão excluídos para evitar que alterações patológicas advindas da obesidade alterem as respostas do estudo.  4.2.3 Índice de qualidade do sono  Para a avaliação da qualidade do sono será utilizado o Índice de Qualidade do Sono de Pittsburg (IQSP). A pontuação máxima desse instrumento é de 21 pontos. Indivíduos que apresentarem escores superiores a 5 pontos tem qualidade ruim de sono (8) e, portanto, serão excluídos, pois a má qualidade do sono pode alterar as respostas cardiovasculares da função cardiovascular (46).    4.2.4 Avaliação do consumo pico de oxigênio  Para a avaliação da condição de saúde cardiovascular, medição do consumo pico de oxigênio e cálculo da potência que será utilizada nas sessões experimentais, os voluntários serão submetidos a um teste ergoespirométrico máximo, realizado em cicloergômetro (Corival Cycle) até a exaustão.  Os indivíduos serão instruídos a fazer uma refeição leve duas horas antes do teste e a não ingerir, neste dia, nenhum alimento que contiver cafeína. Além disso, serão instruídos a não realizar exercícios físicos nas 48 horas que antecederem o teste, e a não ingerirem álcool nas 24h precedentes. O teste será precedido pela execução de um eletrocardiograma de repouso (Cardioperfect, MD) com o registro simultâneo das 12 derivações padrão (D1, D2, D3, aVL, aVF, aVR, V1, V2, V3, V4, V5, V6). Será aplicado um protocolo escalonado com incremento de carga de 30 Watts a cada 3 minutos até a exaustão. Os critérios adotados para a interrupção dos testes serão os seguintes: cansaço físico intenso (caracterizado pela impossibilidade do voluntário em manter a velocidade de 60 rotações por minuto), problemas de saúde que impeçam a continuidade do teste como, por exemplo, PA sistólica maior que 260mmHg e/ou diastólica maior que 120mmHg ou ainda alterações eletrocardiográficas relacionadas a doenças cardiovasculares, seguindo-se as recomendações das III Diretrizes da Sociedade Brasileira de Cardiologia Sobre Teste Ergométrico (2003) (1), e durante os testes, a freqüência cardíaca e a onda eletrocardiográfica serão continuamente monitoradas e registradas ao final dos três minutos de repouso sentado e a cada 3 minutos durante o teste. A PA será aferida pelo método auscultatório, utilizando-se um esfigmomanômetro de coluna de mercúrio, imediatamente antes do registro da freqüência cardíaca. O consumo de oxigênio será continuamente medido a cada ciclo respiratório por um analisador de gases computadorizado (Medical Graphics Corporation, CPX/D).  A análise da condição de saúde dos voluntários será feita por um médico com base em exame clínico e na análise dos eletrocardiogramas de repouso e exercício. Serão excluídos, os voluntários que apresentarem alterações clínicas e/ou eletrocardiográficas sugestivas de problemas cardiovasculares.  A potência aeróbia máxima será avaliada pelo consumo de pico de oxigênio (VO_2_pico), identificado pelo valor mais alto do consumo de oxigênio atingido durante o teste, em média de 30 segundos. Serão excluídos, os indivíduos que praticarem atividade física regularmente (mais de 2 vezes por semana) ou que atingirem uma potência aeróbica máxima acima de 120% do esperado, o que será calculado através da fórmula proposta por Wasserman, onde: VO_2_pico estimado (ml.kg^–1^ min^–1^) = Peso * (50,72 – 0,372) /1000.    4.3 MEDIDAS  4.3.1 PA clínica  Durante as sessões experimentais, a PA clínica será medida por 2 técnicas simultaneamente. No braço dominante, a PA será medida pelo método auscultatório, empregando-se um esfigmomanômetro de coluna de mercúrio, e definindo-se as fases I e V de Korotkoff para a identificação da PA sistólica e diastólica, respectivamente. Essa medida será realizada pelo mesmo observador em todas as sessões experimentais. A PA média será calculada pela soma da PA diastólica com 1/3 da pressão de pulso.  No braço não dominante, a PA será medida batimento a batimento pela técnica fotopletismográfica através do equipamento Finometer (FMS – Finapress Mensurament System, Arnhem, Netherlands) (Elvan-Taspinar, 2003). Para tanto, um manguito inflável de tamanho apropriado será posicionado no dedo médio dos voluntários. Este manguito comporta um sensor infravermelho conectado a uma caixa que fica acoplada ao punho. Essa inclui um sistema controlador rápido de pressão para um ajuste contínuo do manguito de acordo com as mudanças mostradas pelo sensor. O manguito e a caixa do sensor infravermelho serão conectados a uma unidade principal que controla o fluxo de ar. Para a obtenção da onda de PA, o sensor infravermelho perceberá o diâmetro da artéria, enquanto que o sistema de fluxo de ar manterá este diâmetro constante a partir de inflação ou desinflação do manguito. A onda de PA derivará da pressão necessária em cada momento para manter o diâmetro da artéria fixo. A onda de PA obtida por este equipamento será digitalizada e registrada em microcomputador pelo programa Windaq (DI–720) com uma freqüência de amostragem de 500Hz.  4.3.2 Medida ambulatorial da PA  Logo após o término da sessão experimental, a medida oscilométrica da PA será realizada no braço não dominante do voluntário para a avaliação ambulatorial da PA através de um monitor automático (SpaceLabs, 90207), que será programado para realizar medidas a cada 15 minutos por 24 horas. A calibração do equipamento será regularmente checada em comparação com a coluna de mercúrio.  4.3.3 Frequência cardíaca  A freqüência cardíaca será monitorada por um eletrocardiógrafo (TEB-M10) e a onda eletrocardiográfica será digitalizada e registrada em microcomputador pelo programa WINDAQ (DI-720), com uma freqüência de amostragem de 500Hz/canal.    4.3.4 Débito cardíaco  O débito cardíaco será medido pelo método indireto de Fick, através do processo de reinalação de CO_2_ (14, 66, 68). DC = VCO_2_ / (CvCO_2_ – CaCO_2_)  Em que: DC = débito cardíaco, VCO_2_ = produção de dióxido de carbono, CvCO_2_ = conteúdo venoso de CO_2,_ CaCO_2_ = conteúdo arterial de CO_2_.  Para esta análise, será utilizado um analisador de gases computadorizado (Medical Graphics Corporation, CPX/D). Inicialmente, o voluntário irá inspirar normalmente o ar ambiente. Durante este período, o VCO_2_ será medido e o CaCO_2_ será estimado através da medida do PetCO_2_ (pressão expirada final de CO_2_). Em seguida, para avaliar o CvCO_2,_ será realizado o procedimento de reinalação de CO_2_, durante o qual o voluntário irá inspirar e expirar, por um período de 20 segundos, o ar de uma bolsa contendo concentrações de CO_2_ entre 8 e 11% e de O_2_ em 35%. Quando houver o equilíbrio na concentração de CO_2_ na bolsa, o CvCO_2_ será estimado. A partir desses dados, o débito cardíaco será calculado.  4.3.5 Resistência vascular periférica  A resistência vascular periférica (RVP) será calculada pelo quociente entre os valores de PA média auscultatória (PAM) e do débito cardíaco (DC). RVP = PAM / DC.  4.3.6 Volume sistólico  O volume sistólico (VS) será calculado pelo quociente entre o débito cardíaco (DC) e a freqüência cardíaca (FC). VS = DC / FC.  4.3.7 Respiração  Os movimentos respiratórios serão monitorados por uma cinta piezoelétrica (Pneumotrace 2, UFI) posicionada no tórax do voluntário. O sinal respiratório será amplificado (Bioamplifier, 2121/1-R), digitalizado e registrado em microcomputador através do programa WINDAQ (DI 720), com uma freqüência de amostragem de 500 Hz/canal.  4.3.8 Modulação autonômica do sistema cardiovascular  As modulações simpáticas e parassimpáticas do sistema cardiovascular serão avaliadas pela análise espectral da variabilidade da freqüência cardíaca e da PA. Neste método, as ondas eletrocardiográficas, da respiração e da PA, que forem gravadas pelo sistema WINDAQ (DI 720) serão inicialmente analisadas pelo programa PRE.EXE (Programa de Cálculo do Sinal da Variabilidade - versão 3.2), que fornecerá os valores de intervalo R-R, da PA sistólica, da PA diastólica e da respiração a cada ciclo cardíaco, gerando quatro séries temporais.  Posteriormente, a variabilidade destes sinais será analisada no domínio da freqüência pelo método de análise Auto Regressiva (AR), utilizando o software LA (Programa de Análise Linear - versão 8.3). Resumidamente, em seguimentos estacionários das séries temporais, os parâmetros auto-regressivos serão estimados pelo recurso de Levingson-Durbin e a ordem do modelo será escolhida pelo critério de Akaike. Com este procedimento, será possível calcular o poder espectral total e quantificar a freqüência central e o poder de cada componente relevante do espectro, tanto em unidades absolutas, quanto normalizadas.  Para se avaliar a relação da variabilidade da freqüência cardíaca e da PA com a da respiração será realizada a coerência entre os picos de alta e baixa freqüência da variabilidade destes sinais. Serão considerados picos de alta freqüência, aqueles obtidos entre as freqüências de 0,15Hz e 0,4Hz e que apresentarem coerência acima de 0,50 com a respiração. Na ocorrência de mais de um pico com estas características, os poderes espectrais serão somados, e o valor final será definido como o poder da banda de alta freqüência. Serão considerados picos de baixa freqüência, aqueles observados entre 0,04Hz e 0,15Hz e que não apresentarem coerência superior a 0,50 com a respiração. No caso de mais de um pico apresentar estas condições, os poderes espectrais destes picos serão somados para definir a banda de baixa freqüência. O processo de normalização de cada banda de freqüência será realizado dividindo-se o valor da banda pelo valor total do espectro, do qual será subtraído o valor da banda de muito baixa freqüência (<0,04Hz). O resultado será, então, multiplicado por 100. Todos os programas que serão utilizados nesta análise foram desenvolvidos pelo engenheiro Prof. Dr. Alberto Porta da Universidade de Milão, Itália. Esta análise segue as recomendações do “Task Force” sobre o assunto (63).  Para a interpretação dos resultados, todas as variáveis serão consideradas. Os valores absolutos e normalizados da banda de alta freqüência da variabilidade da freqüência cardíaca serão considerados por representar a modulação parassimpática cardíaca, enquanto o valor normalizado da variabilidade da freqüência cardíaca na banda de baixa freqüência será interpretado como decorrente, principalmente, da modulação simpática para o coração. A razão entre as bandas de alta e baixa frequência será considerada como o balanço simpatovagal cardíaco. A modulação simpática vasomotora será considerada pela análise dos valores absolutos da banda de baixa freqüência da variabilidade da PA sistólica e diastólica (63).  4.3.9 Avaliação do controle barorreflexo  Com o intuito de se obter informações sobre a relação entre as alterações da PA e da frequência cardíaca, a sensibilidade espontânea do barorreflexo será avaliada pelo coeficiente alfa, que será calculado pela raiz quadrada do quociente entre os poderes espectrais das bandas de baixa freqüência do intervalo cardíaco e da PA sistólica (54). Para tanto, será necessário haver uma coerência maior que 0,5 entre os picos utilizados para o cálculo e a fase entre eles deverá ser negativa, indicando que a resposta da PA antecede a do intervalo R-R.  4.3.10 Dosagens plasmáticas  Para a dosagem de norepinefrina e epinefrina, o sangue será coletado em um tubo especial, pré-congelado contendo 120 uL de solução EGTA/GSH (glutationa reduzida). A noradrenalina será dosada pelo sistema HPLC com detecção eletroquímica (0,5 volts) (HPLC-ED).  Quanto a dosagem plasmática de vasopressina, serão coletados mais 5 ml de sangue venoso em tubo com 2 gotas de heparina. A técnica utilizada para a dosagem será o radioimunoensaio, usando Kit arginina vasopressina, método RIA da Geneses (Webster, Texas, USA) e equipamento Wizard Automatic Gama Counter 1470 (Meriden, Connecticut, USA).  Para a dosagem de angiotensina II, serão coletados 10 mL de sangue em um tubo resfriado contendo inibidores de protease (0.01 mmol/L p-hydroxy-mercury benzoate; 1.5 mmol/L o-phenanthroline; 0.01 mmol/L para-methyl sulphonil fluoride; 0.05 mmol/L Pepstatin A, and 10 mmol/L ethylenediaminetetraacidic acid) (50 μL por 1 mL de sangue). A concentração de AG II será determinada por radioimunoensaio utilizando um anticorpo específico (Rabbit polyclonal antibody).  Para a dosagem de melatonina, serão coletados 3 ml de sangue e a análise será realizada através de radioimunoensaio utilizando anti-soro de melatonina.  4.3.11 Fluxo sanguíneo  O Fluxo sanguíneo da musculatura ativa (perna) e inativa (braço) será medido pela técnica de pletismografia de oclusão venosa (72). Para tanto, o fluxo será medido no antebraço e na perna. As medidas de fluxo sanguíneo no antebraço e na perna serão realizadas enquanto os fluxos arteriais para a mão (caso do antebraço) ou o pé (caso da perna) forem ocluídos por manguitos colocados em torno do punho ou do tornozelo, respectivamente, e inflados com uma pressão de 200mmHg. Para as medidas, serão colocados no braço (2cm acima da dobra do cotovelo) e na coxa (2cm abaixo da dobra do joelho), manguitos que serão inflados rapidamente (E20 Rapid Cuff Inflator, AG101 Air Source) durante 10 segundos em níveis subdiastólicos, mas um pouco acima da pressão venosa (40 a 60mmHg) para ocluir o retorno venoso, seguidos de 10 segundos de deflação, completando ciclos de 20 segundos cada. Ao serem inflados, estes manguitos impedirão a saída do fluxo venoso do membro, sem impedir o influxo arterial, o que provocará aumento do volume do antebraço e da perna, proporcionais ao fluxo arterial dessas regiões. Esses aumentos serão percebidos por sensores (“mercury-in-silastic strain gauges”) posicionados na maior circunferência do antebraço ou da perna. Essa medida será realizada por 4 minutos. A média de nove ciclos (excluir-se-á os dois primeiros e o último) será usada para determinar o fluxo sangüíneo basal, que será expresso em mL.100 mL^-1^ de tecido.min^-1^. Os sinais obtidos serão registrados em microcomputador através do software NIVP3 (Hokanson Inc. USA). As condutâncias arteriais do antebraço e da perna serão calculadas pelo quociente entre o fluxo sanguíneo e a pressão arterial média (72) medida imediatamente antes da medida do fluxo no braço pelo método auscultatório.  4.3.15 Função endotelial  Para avaliar a função endotelial será utilizada a técnica de hiperemia reativa à isquemia, previamente validada como indicador da vasodilatação dependente do endotélio (70). Para esta avaliação, após a medida do fluxo basal, os manguitos posicionados no braço e acima do joelho serão insuflados rapidamente até 200 mmHg e essa oclusão será mantida por 5 minutos. Durante o último minuto de oclusão arterial, o paciente realizará movimentos com as mãos e com os pés para potencializar o efeito da isquemia sobre o endotélio. Em seguida, os manguitos serão desinflados e os fluxos sanguíneos do antebraço e da perna serão registrados por três minutos, como descrito anteriormente.  A função endotelial será avaliada de 2 formas: 1)pelo valor do fluxo da primeira onda registrada após a desinflação do manguito arterial e 2) pela área sob a curva dos fluxos medidos pós-isquemia até este retornar ao basal (70).  **4.4 INTERVENÇÕES**  4.4.1 Controle  Na sessão controle, os indivíduos serão posicionados na bicicleta ergométrica e nela permanecerão por um período de 60 minutos. Este período é exatamente o mesmo da sessão de exercício aeróbico.  4.4.2 Exercício Aeróbico  Na sessão de exercício físico, este será realizado da seguinte maneira: os indivíduos serão posicionados na bicicleta ergométrica e, permanecerão 7 minutos em repouso. Em seguida, farão 3 minutos de aquecimento (potência equivalente a 50% da potência do exercício), 45 minutos de exercício aeróbico em 50% do VO_2_pico, 3 minutos de recuperação ativa (metade da potência do exercício no 1° minuto, ¼ no segundo e potência 0 no 3° minuto) e 2 minutos de recuperação passiva. A potência correspondente a 50% do VO_2_pico será calculada pela análise de regressão linear entre os valores de potência e VO_2_ medidos no teste ergoespirométrico máximo realizado na avaliação preliminar. Além disso, durante o experimento, o VO_2_ será medido por um analisador de gases computadorizado (Medical Graphics Corporation, CPX/D), para verificar a intensidade real do exercício.  **4.5 PROTOCOLO EXPERIMENTAL**  A Figura 1 apresenta a sequência do protocolo experimental.  Os voluntários assinarão um Termo de Consentimento Livre e Esclarecido e, então, serão submetidos às avaliações preliminares (diagnóstico de PA alterada, diagnóstico de obesidade, qualidade do sono e avaliação ergoespirométrica). A partir dos resultados destas avaliações, serão incluídos no estudo somente aqueles que apresentarem os critérios previamente estabelecidos para a amostra: PA alterada (pré-hipertenso), não serem obesos, não possuírem qualidade de sono ruim, não sofrerem de problemas cardiovasculares e não praticarem atividade física regular. O protocolo experimental será ser iniciado pelo menos três dias após o teste ergoespirométrico.  **Figura 1. Representação Esquemática do Protocolo Experimental**    Durante o protocolo experimental, todos os indivíduos participarão das quatro sessões experimentais, realizadas em ordem aleatória, com intervalo mínimo de três dias entre elas. Duas sessões serão realizadas de manhã e duas no final da tarde. Em cada período do dia serão realizadas 1 sessão controle (C) e 1 de exercício (E). Para cada sessão, será solicitado aos voluntários que compareçam ao laboratório com roupas adequadas para a prática de exercício físico. Eles serão instruídos a não ingerirem álcool nas 24h anteriores, e a evitarem a execução de exercícios físicos nas 48 horas precedentes. Em todas as sessões experimentais, as janelas do laboratório permanecerão fechadas com o intuito de evitar influência da luz externa, a temperatura será mantida entre 22ºC e 24ºC e a luminosidade será de ~300 lux.  O Desenho Experimental das sessões está apresentado na Figura 2.  O protocolo adotado nas sessões experimentais segue a linha de diversos estudos anteriores do laboratório. Nas sessões que terão início pela manhã, os voluntários deverão chegar ao laboratório às 7 horas, e no fim da tarde, os experimentos terão início às 17 horas. Em todas as sessões, os voluntários deverão comparecer em jejum de pelo menos 5 horas e receberão uma refeição padronizada, composta por duas barras de cereais um suco de 50 ml e 100 ml de água. O alimento deverá ser consumido entre 10 e 15 minutos. Em seguida, após esvaziar a bexiga os voluntários permaneceram sentados e em repouso até completarem 60 minutos. Aos 45 minutos será feita a coleta de sangue para a dosagem plasmática de norepinefrina, epinefrina, vasopressina, angiotensina II e melatonina. Na sequência, o voluntário poderá esvaziar a bexiga novamente e retornar ao laboratório, quando os equipamentos serão posicionados e o sujeito permanecerá em repouso até completar os 15 minutos restantes.  Iniciar-se-á, então, o período de pré-intervenção, no qual os voluntários permanecerão sentados em repouso por mais 60 minutos. Neste período, as ondas eletrocardiográficas, da respiração e da PA batimento a batimento serão gravadas por 10 minutos para posterior avaliação da modulação autonômica pela técnica da análise espectral, conforme descrito anteriormente. Posteriormente serão realizadas, em triplicata, as medidas da PA auscultatória, frequência cardíaca e débito cardíaco, e logo após, serão realizadas as medidas de fluxo e reatividade vascular da musculatura ativa (pernas) e inativa (braços).  Após o período de pré-intervenção, os indivíduos se deslocarão para o ciclo ergômetro, no qual permanecerão por 60 minutos, seguindo os procedimentos descritos nos itens 4.4.1 na sessão controle e 4.4.2 na sessão exercício. Em todas as sessões, durante as intervenções, as ondas eletrocardiográficas de respiração e de PA serão gravadas dos 30 aos 40 minutos. A freqüência cardíaca, a PA auscultatória e o débito cardíaco serão medidos em triplicata dos 40 aos 55 minutos. Nestes momentos, o VO_2_ também será medido para se avaliar a intensidade real do exercício.  Imediatamente após o período de intervenção, os indivíduos retornarão a posição sentada e iniciar-se-á o período pós-intervenção. Nos primeiros 10 minutos, será feita uma medida de PA auscultatória. Em seguida, entre os minutos 10 e 20 e dos 35 aos 45 serão registradas as ondas eletrocardiográficas da respiração e da PA batimento a batimento para realização da análise espectral. Além disso, dos 20 aos 30 e dos 50 aos 60 minutos, serão realizados em triplicata, as medidas da PA auscultatória, freqüência cardíaca e débito cardíaco. Para finalizar, dos 60 aos 80 minutos, serão realizadas as medidas do fluxo e, a reatividade vascular da perna e do braço concomitantemente.  No final da sessão, os instrumentos serão desconectados e o voluntário poderá tomar banho. Em seguida, ele retornará ao laboratório e será colocado o monitor ambulatorial da PA, que permanecerá no braço não dominante do voluntário até o dia seguinte. O indivíduo deverá anotar suas atividades e horários no dia que estiver com o monitor, e será instruído a não ingerir bebidas alcoólicas, não fazer exercícios físicos, a não dormir no período diurno e a não tomar banho enquanto estiver com o aparelho. Ele deverá manter os horários e as atividades semelhantes após as quatro sessões experimentais.  Fig. 2 – Sessão experimental  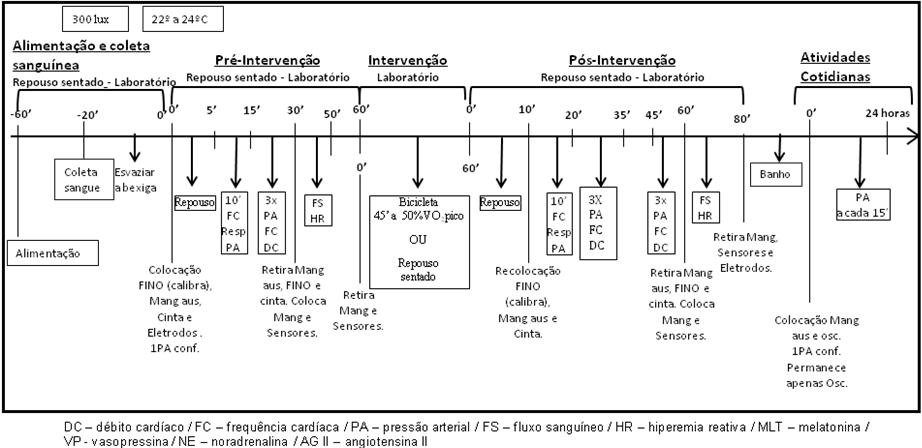  **4.6 ANÁLISES ESTATÍSTICAS**  O s valores das variáveis hemodinâmicas (PA auscultatória, débito cardíaco, freqüência cardíaca, volume sistólico e resistência vascular periférica) medidos em triplicata em cada momento serão calculados pela média das três medidas realizadas.  Quanto aos resultados da monitorização ambulatorial da PA (MAPA), só serão aceitos os registros com pelo menos 85% das medidas válidas (9). Os dados serão avaliados pela média dos valores medidos no período de 24 horas, vigília e sono, bem como, pelas médias horárias após a finalização do exercício.  Todas as variáveis serão testadas quanto à normalidade de sua distribuição pelo teste de Shapiro-Wilkins. Aquelas que não apresentarem distribuição normal sofreram uma transformação logarítmica visando obter a normalidade.  Os dados hemodinâmicos e autonômicos serão analisados pela análise de variância ANOVA de 3 fatores para medidas repetidas, tendo como valores principais: o horário (manhã e tarde), a sessão (controle e exercício) e o estágio (pré e pós-intervenção).  Os dados da MAPA serão comparados pela ANOVA de 2 ou 3 fatores para amostras repetidas, tendo como fatores principais: o horário (manhã e tarde) e a sessão (controle e exercício) e, quando as médias horárias forem analisadas, elas serão consideradas como o terceiro fator.  Quando necessário, será empregado o teste de “post-hoc” de Newman Keuls. O índice de P<0,05 será aceito como significante.  As diferenças de comportamento observadas após o exercício entre o período da manhã e o período da tarde serão correlacionadas com as diferenças observadas nos níveis hormonais medidos nesses dois períodos do dia pela correlação de Pearson ou Spearman dependendo da distribuição normal ou não dos dados. |
| --- |

**5. Referências**

| 1. **Aikawa T, Kasahara T, and Uchiyama M.** Circadian variation of plasma arginine vasopressin concentration, or arginine vasopressin in enuresis. *Scand J Urol Nephrol Suppl* 202: 47-49, 1999.  2. **Aires MM.** *Fisiologia*. Rio de Janeiro: Guanabara Koogan, 2008.  3. **Anwar MM, Meki AR, and Rahma HH.** Inhibitory effects of melatonin on vascular reactivity: possible role of vasoactive mediators. *Comp Biochem Physiol C Toxicol Pharmacol* 130: 357-367, 2001.  4. **Araujo EA.** *Respostas Hemodinâmicas e Autonômicas Pós-Exercício: Influência da Massa Muscular, da Intensidade Relativa e do Gasto Energético Total do Exercício*. São Paulo: Universidade de São Paulo, 2007.  5. **Atkinson G, Drust B, Reilly T, and Waterhouse J.** The relevance of melatonin to sports medicine and science. *Sports Med* 33: 809-831, 2003.  6. **Bertinieri G, di Rienzo M, Cavallazzi A, Ferrari AU, Pedotti A, and Mancia G.** A new approach to analysis of the arterial baroreflex. *J Hypertens Suppl* 3: S79-81, 1985.  7. **Bisquolo VA, Cardoso CG, Jr., Ortega KC, Gusmao JL, Tinucci T, Negrao CE, Wajchenberg BL, Mion D, Jr., and Forjaz CL.** Previous exercise attenuates muscle sympathetic activity and increases blood flow during acute euglycemic hyperinsulinemia. *J Appl Physiol* 98: 866-871, 2005.  8. **Buysse DJ, Reynolds CF, 3rd, Monk TH, Berman SR, and Kupfer DJ.** The Pittsburgh Sleep Quality Index: a new instrument for psychiatric practice and research. *Psychiatry Res* 28: 193-213, 1989.  9. **Cardiologia SBd, Hipertensão SBd, and Nefrologia SBd.** IV Guideline for ambulatory blood pressure monitoring. II Guideline for home blood pressure monitoring. IV ABPM/II HBPM. *Arquivos Brasileiros de Cardiologia* 85: 1-18, 2005.  10. **Cardoso CG, Jr., Gomides RS, Queiroz AC, Pinto LG, da Silveira Lobo F, Tinucci T, Mion D, Jr., and de Moraes Forjaz CL.** Acute and chronic effects of aerobic and resistance exercise on ambulatory blood pressure. *Clinics (Sao Paulo)* 65: 317-325.  11. **Casonato J PM, D.** Hipotensão pós-exercício aeróbico: uma revisão sistemática. *Revista Brsileira de Medicina do Esporte* 15: 151-157, 2009.  12. **Chobanian AV, Bakris GL, Black HR, Cushman WC, Green LA, Izzo JL, Jr., Jones DW, Materson BJ, Oparil S, Wright JT, Jr., and Roccella EJ.** Seventh report of the Joint National Committee on Prevention, Detection, Evaluation, and Treatment of High Blood Pressure. *Hypertension* 42: 1206-1252, 2003.  13. **Cleroux J, Kouame N, Nadeau A, Coulombe D, and Lacourciere Y.** Aftereffects of exercise on regional and systemic hemodynamics in hypertension. *Hypertension* 19: 183-191, 1992.  14. **Collier CR.** Determination of mixed venous CO2 tensions by rebreathing. *J Appl Physiol* 9: 25-29, 1956.  15. **Cugini P and Lucia P.** [Circadian rhythm of the renin-angiotensin-aldosterone system: a summary of our research studies]. *Clin Ter* 155: 287-291, 2004.  16. **Davies GJ, Chierchia S, and Maseri A.** Prevention of myocardial infarction by very early treatment with intracoronary streptokinase. Some clinical observations. *N Engl J Med* 311: 1488-1492, 1984.  17. **Dimitrov S, Benedict C, Heutling D, Westermann J, Born J, and Lange T.** Cortisol and epinephrine control opposing circadian rhythms in T cell subsets. *Blood* 113: 5134-5143, 2009.  18. **Dujic Z, Ivancev V, Valic Z, Bakovic D, Marinovic-Terzic I, Eterovic D, and Wisloff U.** Postexercise hypotension in moderately trained athletes after maximal exercise. *Med Sci Sports Exerc* 38: 318-322, 2006.  19. **Fagard RH.** Effects of exercise, diet and their combination on blood pressure. *J Hum Hypertens* 19 Suppl 3: S20-24, 2005.  20. **Forjaz CL, Cardoso CG, Jr., Rezk CC, Santaella DF, and Tinucci T.** Postexercise hypotension and hemodynamics: the role of exercise intensity. *J Sports Med Phys Fitness* 44: 54-62, 2004.  21. **Forjaz CL, Matsudaira Y, Rodrigues FB, Nunes N, and Negrao CE.** Post-exercise changes in blood pressure, heart rate and rate pressure product at different exercise intensities in normotensive humans. *Braz J Med Biol Res* 31: 1247-1255, 1998.  22. **Forjaz CL, Ramires PR, Tinucci T, Ortega KC, Salomao HE, Ignes EC, Wajchenberg BL, Negrao CE, and Mion D, Jr.** Postexercise responses of muscle sympathetic nerve activity and blood flow to hyperinsulinemia in humans. *J Appl Physiol* 87: 824-829, 1999.  23. **Forjaz CL, Santaella DF, Rezende LO, Barretto AC, and Negrao CE.** [Effect of exercise duration on the magnitude and duration of post-exercise hypotension]. *Arq Bras Cardiol* 70: 99-104, 1998.  24. **Forjaz CL, Tinucci T, Ortega KC, Santaella DF, Mion D, Jr., and Negrao CE.** Factors affecting post-exercise hypotension in normotensive and hypertensive humans. *Blood Press Monit* 5: 255-262, 2000.  25. **Grassi G, Bombelli M, Seravalle G, Dell'Oro R, and Quarti-Trevano F.** Diurnal blood pressure variation and sympathetic activity. *Hypertens Res* 33: 381-385.  26. **Hagberg JM, Montain SJ, and Martin WH, 3rd.** Blood pressure and hemodynamic responses after exercise in older hypertensives. *J Appl Physiol* 63: 270-276, 1987.  27. **Halliwill JR.** Mechanisms and clinical implications of post-exercise hypotension in humans. *Exerc Sport Sci Rev* 29: 65-70, 2001.  28. **Halliwill JR, Taylor JA, and Eckberg DL.** Impaired sympathetic vascular regulation in humans after acute dynamic exercise. *J Physiol* 495 ( Pt 1): 279-288, 1996.  29. **Halliwill JR, Taylor JA, Hartwig TD, and Eckberg DL.** Augmented baroreflex heart rate gain after moderate-intensity, dynamic exercise. *Am J Physiol* 270: R420-426, 1996.  30. **Hamer M and Boutcher SH.** Impact of moderate overweight and body composition on postexercise hemodynamic responses in healthy men. *J Hum Hypertens* 20: 612-617, 2006.  31. **Harvey PJ, Morris BL, Kubo T, Picton PE, Su WS, Notarius CF, and Floras JS.** Hemodynamic after-effects of acute dynamic exercise in sedentary normotensive postmenopausal women. *J Hypertens* 23: 285-292, 2005.  32. **Hvistendahl GM, Frokiaer J, Nielsen S, and Djurhuus JC.** Gender differences in nighttime plasma arginine vasopressin and delayed compensatory urine output in the elderly population after desmopressin. *J Urol* 178: 2671-2676, 2007.  33. **Jones H, George K, Edwards B, and Atkinson G.** Effects of time of day on post-exercise blood pressure: circadian or sleep-related influences? *Chronobiol Int* 25: 987-998, 2008.  34. **Jones H, George K, Edwards B, and Atkinson G.** Is the magnitude of acute post-exercise hypotension mediated by exercise intensity or total work done? *Eur J Appl Physiol* 102: 33-40, 2007.  35. **Jones H, Pritchard C, George K, Edwards B, and Atkinson G.** The acute post-exercise response of blood pressure varies with time of day. *Eur J Appl Physiol* 104: 481-489, 2008.  36. **Jones H, Taylor CE, Lewis NC, George K, and Atkinson G.** Post-exercise blood pressure reduction is greater following intermittent than continuous exercise and is influenced less by diurnal variation. *Chronobiol Int* 26: 293-306, 2009.  37. **Kawano H, Motoyama T, Yasue H, Hirai N, Waly HM, Kugiyama K, and Ogawa H.** Endothelial function fluctuates with diurnal variation in the frequency of ischemic episodes in patients with variant angina. *J Am Coll Cardiol* 40: 266-270, 2002.  38. **Kenney MJ and Seals DR.** Postexercise hypotension. Key features, mechanisms, and clinical significance. *Hypertension* 22: 653-664, 1993.  39. **Kitajima T, Kanbayashi T, Saitoh Y, Ogawa Y, Sugiyama T, Kaneko Y, Sasaki Y, Aizawa R, and Shimisu T.** The effects of oral melatonin on the autonomic function in healthy subjects. *Psychiatry Clin Neurosci* 55: 299-300, 2001.  40. **Legramante JM, Galante A, Massaro M, Attanasio A, Raimondi G, Pigozzi F, and Iellamo F.** Hemodynamic and autonomic correlates of postexercise hypotension in patients with mild hypertension. *Am J Physiol Regul Integr Comp Physiol* 282: R1037-1043, 2002.  41. **Lewis NC, Atkinson G, Lucas SJ, Grant EJ, Jones H, Tzeng YC, Horsman H, and Ainslie PN.** Diurnal variation in time to presyncope and associated circulatory changes during a controlled orthostatic challenge. *Am J Physiol Regul Integr Comp Physiol* 299: R55-61, 2010.  42. **Lewis NC, Atkinson G, Lucas SJ, Grant EJ, Jones H, Tzeng YC, Horsman H, and Ainslie PN.** Diurnal variation in time to presyncope and associated circulatory changes during a controlled orthostatic challenge. *Am J Physiol Regul Integr Comp Physiol* 299: R55-61.  43. **Lockwood JM, Pricher MP, Wilkins BW, Holowatz LA, and Halliwill JR.** Postexercise hypotension is not explained by a prostaglandin-dependent peripheral vasodilation. *J Appl Physiol* 98: 447-453, 2005.  44. **Lockwood JM, Wilkins BW, and Halliwill JR.** H1 receptor-mediated vasodilatation contributes to postexercise hypotension. *J Physiol* 563: 633-642, 2005.  45. **Lombardi F, Sandrone G, Mortara A, La Rovere MT, Colombo E, Guzzetti S, and Malliani A.** Circadian variation of spectral indices of heart rate variability after myocardial infarction. *Am Heart J* 123: 1521-1529, 1992.  46. **Lynn BM, Minson CT, and Halliwill JR.** Fluid replacement and heat stress during exercise alter post-exercise cardiac haemodynamics in endurance exercise-trained men. *J Physiol* 587: 3605-3617, 2009.  47. **M DEZ, Covassin N, G DEMT, Sarlo M, and Stegagno L.** Sleep onset and cardiovascular activity in primary insomnia. *J Sleep Res*.  48. **MacDonald JR.** Potential causes, mechanisms, and implications of post exercise hypotension. *J Hum Hypertens* 16: 225-236, 2002.  49. **McCord JL, Beasley JM, and Halliwill JR.** H2-receptor-mediated vasodilation contributes to postexercise hypotension. *J Appl Physiol* 100: 67-75, 2006.  50. **Millar-Craig MW, Bishop CN, and Raftery EB.** Circadian variation of blood-pressure. *Lancet* 1: 795-797, 1978.  51. **Nakagawa H and Okumura N.** Coordinated regulation of circadian rhythms and homeostasis by the suprachiasmatic nucleus. *Proc Jpn Acad Ser B Phys Biol Sci* 86: 391-409.  52. **NIH NIoH.** The Practical Guide Identification, Evaluation, and Treatment of Overweight and Obesity in Adults. *NIH Publicatio*: 1-75, 2000.  53. **Notarius CF, Morris BL, and Floras JS.** Caffeine attenuates early post-exercise hypotension in middle-aged subjects. *Am J Hypertens* 19: 184-188, 2006.  54. **Otto ME, Svatikova A, Barretto RB, Santos S, Hoffmann M, Khandheria B, and Somers V.** Early morning attenuation of endothelial function in healthy humans. *Circulation* 109: 2507-2510, 2004.  55. **Pagani M, Somers V, Furlan R, Dell'Orto S, Conway J, Baselli G, Cerutti S, Sleight P, and Malliani A.** Changes in autonomic regulation induced by physical training in mild hypertension. *Hypertension* 12: 600-610, 1988.  56. **Panza JA, Epstein SE, and Quyyumi AA.** Circadian variation in vascular tone and its relation to alpha-sympathetic vasoconstrictor activity. *N Engl J Med* 325: 986-990, 1991.  57. **Park SW, Choi SM, and Lee SM.** Effect of melatonin on altered expression of vasoregulatory genes during hepatic ischemia/reperfusion. *Arch Pharm Res* 30: 1619-1624, 2007.  58. **Pescatello LS, Franklin BA, Fagard R, Farquhar WB, Kelley GA, and Ray CA.** American College of Sports Medicine position stand. Exercise and hypertension. *Med Sci Sports Exerc* 36: 533-553, 2004.  59. **Rondon MUPB, Alves MJNN, Braga AMFW, Teixeira OTUN, Barreto ACP, Krieger EM, and Negrão CE.** Postexercise Blood Pressure Reduction in Elderly Hypertensive Patients. *Journal of the American College of Cardiology* 39: 676-682, 2002.  60. **SBd. H.** VI Diretrizes Brasileiras de Hipertensão. *Revista Hipertensão* 13: 1-68, 2010.  61. **Scheer FA, Kalsbeek A, and Buijs RM.** Cardiovascular control by the suprachiasmatic nucleus: neural and neuroendocrine mechanisms in human and rat. *Biol Chem* 384: 697-709, 2003.  62. **Scott JM, Esch BT, Lusina SJ, McKenzie DC, Koehle MS, Sheel AW, and Warburton DE.** Post-exercise hypotension and cardiovascular responses to moderate orthostatic stress in endurance-trained males. *Appl Physiol Nutr Metab* 33: 246-253, 2008.  63. **Senitko AN, Charkoudian N, and Halliwill JR.** Influence of endurance exercise training status and gender on postexercise hypotension. *J Appl Physiol* 92: 2368-2374, 2002.  64. **Task Force of the European Society of Cardiology and the North American Society of Pacing and Electrophysiology.** Heart rate variability: standards of measurement, physiological interpretation and clinical use. Task Force of the European Society of Cardiology and the North American Society of Pacing and Electrophysiology. *Circulation* 93: 1043-1065, 1996.  65. **Teixeira L.** *Efeito isolado e associado do exercício aeróbio e resistido na pressão arterial pós-exercício e seus mecanismos hemodinâmicos, neurais e de estado de ansiedade* (Mestrado). São Paulo: Universidade de São Paulo, 2007.  66. **Tochikubo O, Kawano Y, Miyajima E, Toshihiro N, and Ishii M.** Circadian variation of hemodynamics and baroreflex functions in patients with essential hypertension. *Hypertens Res* 20: 157-166, 1997.  67. **Turner MJ, Tanaka H, Bassett DR, Jr., and Fitton TR.** The equilibrium CO2 rebreathing method does not affect resting or exercise blood pressure. *Med Sci Sports Exerc* 28: 921-925, 1996.  68. **Van de Borne P, Nguyen H, Biston P, Linkowski P, and Degaute JP.** Effects of wake and sleep stages on the 24-h autonomic control of blood pressure and heart rate in recumbent men. *Am J Physiol* 266: H548-554, 1994.  69. **Vanhees L, Defoor J, Schepers D, Brusselle S, Reybrouck T, and Fagard R.** Comparison of cardiac output measured by two automated methods of CO2 rebreathing. *Med Sci Sports Exerc* 32: 1028-1034, 2000.  70. **Vogel RA.** Measurement of endothelial function by brachial artery flow-mediated vasodilation. *Am J Cardiol* 88: 31E-34E, 2001.  71. **Voogel AJ, Koopman MG, Hart AA, van Montfrans GA, and Arisz L.** Circadian rhythms in systemic hemodynamics and renal function in healthy subjects and patients with nephrotic syndrome. *Kidney Int* 59: 1873-1880, 2001.  72. **Wilkinson IB and Webb DJ.** Venous occlusion plethysmography in cardiovascular research: methodology and clinical applications. *Br J Clin Pharmacol* 52: 631-646, 2001. |
| --- |

**6. Apêndices**
