# Supplementary material for: Post-Exercise Hypotension and Its Mechanisms Differ after Morning and Evening Exercise: A Randomized Crossover Study
Source: PLoS One. 2015 Jul 17;10(7):e0132458. doi: 10.1371/journal.pone.0132458 (PMC4506120; doi:10.1371/journal.pone.0132458)
Supplement: S2 File — (DOCX) [file pone.0132458.s003.docx]

# RESEARCH PROJECT

*(no more than 20 pages)*

#### 1. General Information

**Title:** Post-Aerobic Exercise Hypotension and its Hemodynamic and Neural Mechanisms in Pre-Hypertensive Men: influence of time of day and association with circadian endocrine regulation

**Responsible Researcher:** Cláudia Lúcia de Moraes Forjaz, PhD

**Manager Researcher:** Leandro Campos de Brito

**Department Engaged:** Biodynamics of Human Movement

**2. Introduction**

| Regular practice of physical exercise, mainly aerobic, has been recommended as a non-pharmacological therapy for the prevention and treatment of arterial hypertension. Thus, this practice is recommended by national and international guidelines for hypertensive and pre-hypertensive individuals (12, 59). The hypotensive effects of exercise can be observed after a period of training (10, 19). However, it has also been reported after performing a single bout of exercise (11, 21, 23, 24, 27, 47, 57). Indeed, after an aerobic exercise session, blood pressure (BP) decreases below values observed before the exercise or those assessed in a control day without any exercise. This phenomenon has been called post-exercise hypotension (PEH) (38).  PEH has been studied by many research groups, including ours. In 2004, its clinical relevance has been pointed out by the American College of Sports Medicine because its presents significant magnitude and lasts long after the exercise bout (57). A previous study from our group showed that BP decrease may last up to 24 hours after exercise in normotensives (24). Rondon et al (2002) reported similar results in hypertensives. Regarding the magnitude of PEH, MacDonald’ review (2002) reported that systolic/diastolic mean BP decreases range 8/9, 14/9 e 10/7 mmHg in normotensive, borderline and hypertensive subjects, respectively.  Despite of its clinical relevance, PEH magnitude and duration varies a lot among the studies, suggesting that they are affected by many factors. Thus, the current study will investigate the possible effect of circadian rhythm on PEH.  Previous studies from Dr Jones’ group (32, 34-36) suggest that performing an aerobic exercise in the morning promotes no or a lower decrease in BP during recovery than performing the same exercise in the evening. However, these studies did not include a control session without exercise, which would allow assessing BP changes caused by time pass inteady of exercise. This absence might have promoted a misinterpretation of the results, since BP levels usually increase (16, 49) throughout the morning period. Thus, if BP does not increase after performing exercise in the morning, a hypotensive effect of exercise is present and it may be as expressive as a BP decrease observed after evening exercise. Thus, the inclusion of a control session in the study design is very important. Therefore, the first aim of the current study is to assess the influence of time of day on BP responses after exercise controlling for responses observed at these times of day without exercise.  The hemodynamic determinants of PEH have also been studied; however, the results are still inconclusive. Some studies found that BP decrease after exercise was due to a cardiac output decrease (5, 18, 26, 30, 45, 58, 62), while other studies observed a decrease of systemic vascular resistance (13, 20, 27-29, 31, 33, 40, 42, 43, 47, 48, 61, 64). Likewise, many factors can also influence these responses and, in the current study, the possible influence of time of day will be investigated.  When PEH is associated to cardiac output decrease, a decrease in stroke volume promoted by a reduction in pre-load and not compensated despited of the increase in heart rate triggered by the high sympathetic modulation to the heart are considered the underline mechanisms (18, 26, 30, 45, 58, 62). However, a decrease in pre-load deactivates the cardiopulmonary reflex, increasing systemic vascular resistance (3), which does not occur after aerobic exercise, suggesting that exercise has also an effect on peripheral circulation, blunting the systemic vascular resistance increase.  When PEH is accompanied by a decrease in systemic vascular resistance decrease, this decrease is attributed to the maintainance of exercise-induced vasodilation during the recovery period, which can result from the decrease in peripheral sympathetic activity (7, 22), the release of vasodilatory substances (43, 48, 52) or the decrease of vascular responsivity to vasoconstrictor stimulus (28).  Regarding time of day, it is known that hemodynamic variables present circadian patterns. In the morning, after waking up, BP increases in response to the increase in some pressor mechanisms. Sympathetic activity to heart and vessels rises, increasing heart rate (25, 44) and systemic vascular resistance (55). Moreover, at this time of day, baroreflex sensibility decreases (65, 67), which favors BP increase. Likewise, endothelial function is blunted in the morning (37, 53), difficulting vasodilation and favoring the increase of systemic vascular resistance and BP. Thus, since neural, hormonal and hemodynamic variables present circadian patterns, it is possible that reponses of these parameters after exercise are diferent when exercise is performed in the morning and the evening.  Dra Jones’s studies reported that the lower PEH observed in the morning was related to a greater systemic vascular resistance (32, 35). However, these authors did not investigate other hemodynamic parameters, and did not assess neural and hormonal regulation of PEH. Therefore, another objective of this study is to assess hemodynamic and neural mechanisms of PEH after morning and evening exercise.  Circadian patterns observed in different physiologic functions are controlled by a central biologic clock, the suprachiasmatic nucleous. This nucleous has pre-sympathetic and pre-parasympathetic neurons (50), which allows it to integrate the biologic clock with central and peripheric cardiovascular autonomic regulation (60). Contributing to this regulation, the circadian light/dark cycle is marked by melatonin release. This hormone, produced by the pineal gland, achieves levels 30 times greater at night than in the morning (6). It also influences baroreflex control decreasing its set-point (39) and it has vasodilatory properties (4, 56). Thus, the suprachiasmatic nucleous also indirectly influences BP and its hemodynamic mechanisms by controlling melatonin (60).  Other hormones that regulate BP also present circadian patterns. Noradrenaline and angiotensin II increase in the morning (15, 17), while vasopressin decreases (2), explaining the stroke volume behavior at this time of day (41, 70). Thus, another objective of the current study is to observe the relationship between some circadian rhythm hormonal markers and the physiologic responses after exercise performed at different times of day.  According to previous background, the hypothesis of this study is that, in the morning, PEH corrected by changes observed after a control session will be the same or a little bit lower than in the evening. Additionally, in the morning, this response will be accompanied by a lower cardiac output, due to a greater decrease in stroke volume. The decrease in systemic vascular resistance will be mitigated in the morning. Finally, the different responses observed at different times of day will be associated with plasma levels of noradrenaline, angiotensin II, vasopressin and melatonin at each time of day. |
| --- |

**3. General and Specific Objectives**

| **3.1 General**  To analyze the influence of time of day at which exercise is perfomed on hemodynamic, vascular and neural determinants of PEH, relating this influence to the neuro-hormonal characteristics observed at each time of day.  **3.2 Specific**  To compare physiological responses after an aerobic exercise session performed in the morning and the evening:   1. Clinic and 24-hour blood pressure; 2. Hemodynamic determinants: cardiac output, systemic vascular resistance, stroke volume and heart rate; 3. Neural determinants: sympathetic and parasympathetic heart modulation, vasomotor sympathetic modulation and spontaneous baroreflex sensibility; 4. Blood flow and vasodilatory capacity;   To correlate hemodynamic, neural and vascular responses observed after exercise performed in the morning and evening with noradrenaline, adrenaline, angiotensin II and vasopressin plasmatic levels. |
| --- |

**4. Methods**

| **4.1 Sample**  Sixteen pre-hypertensive adult men, aging between 20-45 years old, nonobese, and not practiticing any regular physical exercise. None of the volunteers can take medications that affect the cardiovascular system. The volunteers will be informed about experimental procedures and possible risks involved in the study. Then, they will give written informed consent to participate. This study will be submitted to Ethic Committee in Research of School of Physical Education and Sport of University of São Paulo.  **4.2 Preliminary procedures**  4.2.1 Diagnosis of pre-hypertension  BP will be assessed three times on each arm after resting for five minutes in the sitting position, as suggested by The Seventh Report of the Joint National Committee on Prevention, Detection, Evaluation, and Treatment of High Blood Pressure: the JNC 7report, (2003). Measurements will be repeated in two visits to the laboratory using a mercury column sphygmomanometer, and employing phases I and V of the Korotkoff sounds to identify systolic and diastolic BP, respectively. The mean of six values assessed in each arm will be calculated and the greater value between the arms will be considered for diagnosis. Only subjects with systolic and diastolic BP below 140 and 90 mmHg, respectively, will be included in the study. Moreover, to characterize pre-hypertension, at least one of the BP values will have to be between: systolic BP = 120 and 139 mmHg or diastolic BP = 80 and 89 mmHg.  4.2.2 Diagnosis of obesity  Weight and height will be assessed using a balance (Welmy) and body mass index (BMI) will be calculated by quotient of weight (kg) and the square of height (m^2^). Only subjects with BMI below 30kg/m^2^ will be included in the study (51).  4.2.3 Sleep quality index  Sleep quality will be assessed by Pittsburgh Sleep Quality Index (PSQI). The maximal possible score is 21. Subjects who present score above 5 have bad sleep quality (8) and will be excluded because bad sleep quality can influence cardiovascular responses (46).  4.2.4 Assessemnt of consumption peak of oxygen  To assess cardiovascular health, peak oxygen consumption and to calculate the workload to be used in the experimental sessions, all volunteers will undergo a maximal cardiopulmonary exercise test conducted on a cycle ergometer (Corival Cycle).  Subjects will be instructed to have a light meal two hours before the test and not to ingest caffeine on this day. Moreover, they will be instructed to avoid exercise for the previous 48 hours and alcohol ingestion for the previous 24 hours. A rest electrocardiogram with the 12 standard derivations (D1, D2, D3, aVL, aVF, aVR, V1, V2, V3, V4, V5, V6) will be recorded before the test (Cardioperfect, MD). A protocol of 30 watts increments each 3 minutes will be applied up to exhaustion. Criteria adopted to stop the test will be: physical fatigue (not able to keep the velocity at 60 revolutions per minute) and healthy problems (such as systolic BP greater than 260 mmHg and/or diastolic BP greater than 120 mmHg, electrocardiographic alterations related to heart diseases). These criteria follow the recommendation of the III Guidelines of Brazilian Society of Cardiology of maximal exercise tests (2003) (1). During the test execution, heart rate and electrocardiographic waves will be monitored and recorded at the end of the 3 minutes of sitting rest and at each 3 minutes during the test. BP will be assessed by auscultatory method employing a mercury column sphygmomanometer immediately before heart rate record. Oxygen consumption will be continuously assessed breath by breath by a metabolic card (Medical Graphics Corporation, CPX/D).  Assessement of volunteers’ health will be performed for a physician considering a clinical exam and the electrocardiogram obtained at rest and during exercise. Volunteers who present clinical and/or electrocardiographic alterations suggestive of cardiovascular problems will be excluded. Aerobic power will be considered by the peak oxygen uptake (VO_2_peak) identified as the greater oxygen uptake achieved during the test in 30 s-averages. Volunteers who practice exercise regularly (more than 2 times per week) or who reach aerobic power greater than 120% of the predicted by Wasserman formule [VO_2_peak estimated (ml.kg^–1^ min^–1^) = Weight * (50.72 – 0.372) /1000] will be excluded.  **4.3 MEASURES**  4.3.1 Clinic BP  During the experimental sessions, clinic BP will be simultaneously assessed by two techniques. On the dominant arm, the auscultatory method will be used, employing a mercury column sphygmomanometer and phases I and V of the Korotkoff sounds for defining systolic and diastolic BP, respectively. This assessment will be performed by the same evaluator at all moments and experimental sessions. Mean BP will be calculated by the sum of diastolic BP and 1/3 of pulse pressure. On the non-dominant arm, BP will be assessed by beat-to-beat photoplethysmography, employing the Finometer (FMS – Finapress Mensurament System, Arnhem, Netherlands). A cuff of apropriate size will be put on the volunteer’s mean finger. BP wave obtained will be digitalized and recorded by Windaq (DI–720) with a frequency sample of 500 Hz.  4.3.2 Ambulatory BP  After the experimental sessions, ocillometric ambulatorial BP will be assessed on the volunteer’s non-dominant arm by an automatic device (SpaceLabs, 90207) that will be programmed to take measurements every 15 minutes for 24 hours. The device calibration will be regularly checked in comparison with a mercury column.  4.3.3 Heart rate  Heart rate will be monitored by an electrocardiogram (EMG System do Brazil, EMG 030110/00B, São Paulo, Brazil) and the eletrocardiographic wave will be digitalized and recorded by WINDAQ (DI-720) with a frequency sample of 500 Hz.  4.3.4 Cardiac output  Cardiac output (CO) will be assessed by the Fick’s indirect method, through CO_2_ rebreathing technique (14, 66, 68). This method uses the following formule: CO = VCO_2_ / (CvCO_2_ – CaCO_2_), in which CO = cardiac output, VCO_2_ = carbon dioxide production, CvCO_2_ = CO_2_ venous content, CaCO_2_ = CO_2_ arterial content. For this measure, a metabolic card (Medical Graphics Corporation, CPX/D) will be used. First, the volunteer will normally breathe the environmental air, VCO_2_ will be assessed and CaCO_2_ will be estimated through PetCO_2_. Then, to assess CvCO_2_, the CO_2_ rebreathing procedure will be performed. The volunteer will inhale and expire for 20 seconds the air containing inside a bag with concentrations of CO_2_ between 8 and 11% and O_2_ of 35%. CvCO_2_ will be estimated and CO will be calculated when CO_2_ reaches an equilibrium.  4.3.5 Systemic vascular resistance  Systemic vascular resistance (SVR) will be calculated by the quotient between auscultatory mean BP (MBP) and CO. SVR = MBP / CO.  4.3.6 Stroke volume  Stroke volume (SV) will be calculated by quotient between CO and heart rate (HR). SV = CO / HR.  4.3.7 Breathing  Respiratory movements will be monitored by a piezoelectric belt (Pneumotrace 2, UFI) positioned on the volunteer’s torax. Respiratory signal will be amplified (Bioamplifier, 2121/1-R), digitalized and recorded by the WINDAQ (DI 720), with a sample frequency of 500 Hz/chanel.  4.3.8 Cardiovascular autonomic modulation of system  Sympathetic and parasympathetic modulations of cardiovascular system will be assessed by spectral analysis of HR and BP variabilities. Electrocardiographic, respiratory and BP waves, recorded by WINDAQ (DI 720), will be analyzed by PRE.EXE software (Program of Variability Signals Calculus – version 3.2). This program will provide R-R interval, respiratory, systolic BP and diastolic BP values for each cardiac cycle, creating four time series. Then, the variability of signals will be analyzed in the frequency domain by the Auto Regressive (AR) method, employing the LA software (Program of Linear Analysis – version 8.3). Briefly, in stationary segments of the time series, auto-regressive parameters will be estimated by Levingson-Durbin resource and the model order will be chosen by Akaike criterion. Through this procedure, it will be possible to calculate spectral power of each band of frequence in both absolute and normalized units.  To assess the relationship of HR and BP variabilities with respiration, the coherence of the signal variabilities at high and low frequency bands will be calculate. High frequency peaks will be defined as those between 0.15 and 0.4Hz and with coherence above 0.50. Low frequency peaks will be defined as those between 0.04 and 0.15Hz without coherence with respiration above 0.50. Normalization process of each band will be performed dividing the band’s power by the total power of spectrum, from which the very low frequency (<0.04Hz) will be subtracted, and the result will be multiplied by 100. All softwares used in this analysis have been developed by Dr Alberto Porta, PhD from University of Milan, Italy. This analysis will follow the recommendation of the Task Force (63).  All variables will be considered. Absolute and normalized values of high frequency band of HR variability will be considered as representative of cardiac parasympathetic modulation, while the normalized value of low frequency band of HR variability will be considered for representing mainly cardiac sympathetic modulation. The ratio between low and high frequency bands will be considered as the cardiac sympathovagal balance. Vasomotor sympathetic modulation will be considered by the absolute values of low frequency bands of systolic and diastolic BP variabilities (63).  4.3.9 Assessment of barreflex control  Spontaneous baroreflex sensibility will be assessed by alpha coefficient, which will be calculated by the square root of the quotient between spectral powers of low frequency bands of RR interval and systolic BP (54). A coherence above 0.50 between these peaks and a negative phase between them will be necessary.  4.3.10 Plasma assessments  For noradreline and adrenaline dosage, 5 ml of blood sample will be collected in a tube containing a priorly frozen solution of 120 uL of EGTA/GSH (reduced glutathione). Noradrenaline will be assessed by HPLC system with electrochemistry detection (0.5 volts) (HPLC-ED). To plasma vasopressin assessment, 5 ml of venous blood will be collected in a tube with 2 gouts of heparin. Dosage will be performed by radioimmunoassay using arginin vasopressin Kit, RIA method of Geneses (Webster, Texas, USA) and using the Wizard Automatic Gama Counter 1470 device (Meriden, Connecticut, USA).  To assess angiotensin II (AG II), 10 ml of blood will be collected in a cold tube containing protease inhibitors (0.01 mmol/L p-hydroxy-mercury benzoate; 1.5 mmol/L o-phenanthroline; 0.01 mmol/L para-methyl sulphonil fluoride; 0.05 mmol/L Pepstatin A, and 10 mmol/L ethylenediaminetetraacidic acid) (50 μL por 1 mL de sangue). AG II concentration will be determined by radioimmunoassay using a specific antibody (Rabbit polyclonal antibody).  To assess melatonin, 3 ml of blood will be collected and the analysis will be performed through radioimmunoassay using melatonin antiserum.  4.3.11 Blood flow  Blood flow for the active (leg) and the inactive (arm) musculatures will assessed by venous occlusion plethysmography (71). Briefly, blood flow will be assessed on the forearm and calf, while the arterial flow to the hand and the foot will be occluded by cuffs positioned at the wrist and the ankle, and inflated above 200 mmHg. To execute the measurements, mercury strain gauges will be positioned on the arm (2cm below the antecubital fossa) and on the calf (2cm below the articular capsule of the knee). Cuffs will be quickly inflated (E20 Rapid Cuff Inflator, AG101 Air Source) for 10 seconds at subdiastolic levels and a little bit above venous pressure (from 40 to 60 mmHg) to occlude venous return. Cycles of inflation (10 s) and deflation (10 s) will last 20 seconds and will be repeated 12 times. When inflated, these cuffs block the limb venous return but allow arterial influx, which increases forearm and calf volumes proportionally to the arterial flow of the limb. This increase is perceived by the strain gauges. The mean of 9 cycles will be used to determine baseline blood flow expressed as mL.100 mL^-1^ tissue.min^-1^. Blood flow waves will be recorded by NIVP3 software (Hokanson Inc. USA). Forearm and calf arterial conductance will be calculated by quotient between the blood flow and the auscultatory MBP (71) assessed immediately before assessing blood flow.  .  4.3.15 Vasodilatory capacity  To assess vasodilatory capacity, reactive hyperemia to ischemia will be used (69). After baseline blood flow measurement, the cuffs positioned on the arm and the thigh will be quickly inflated up to 200 mmHg and this occlusion will be kept for 5 minutes. At the last minute of occlusion, the volunteer will perform movements with his hand and foot to potentiate the ischemic effect. Then, the cuffs will be desinflated and the forearm and calf blood flow will be recorded for 4 minutes as aforementioned for baseline blood flow.  The vasodilatory capacity will be analyzed by 2 ways: 1) the first wave value recorded after deflating the cuffs; and 2) the area under the curve of the values measured after the ischemia (69).  **4.4 INTERVENTIONS**  4.4.1 Control  In the control session, volunteers will sit on the cycle ergometer and stay there for 60 minutes. This is the same amount of time of the aerobic exercise.  4.4.2 Aerobic Exercise  The aerobic exercise session will be performed as follows: volunteers will sit on the cycle ergometer and rest for 7 minutes. Then, they will warm up for 3 minutes (workload of 50% of exercise workload), exercise for 45 minutes (50% of VO_2_peak), have an active recovery for 3 minutes (half workload at 1º minute, ¼ at 2º minute and 0 watts at 3º minute), and have an inactive recovery for 2 minutes. The exercise workload will be calculated by the linear regression between workload and VO_2_ dirung the previous maximal cardiopulmonary exercise test. Moreover, during the experimental sessions, VO_2_ will be assessed by a metabolic card (Medical Graphics Corporation, CPX/D) to verify the real intensity of exercise.  **4.5 EXPERIMENTAL PROTOCOL**  Figure 1 presents the sequency of the experimental protocol.  All volunteers who give written informed consent will undergo the preliminary evaluations (diagnosis of pre-hypertension, diagnosis of obesity, sleep quality, and cardiopulmanry exercise test).Volunteers will stay in this study if they fulfill all the study criteria. Pre-hypertension, non obese, good sleep quality, no cardiovascular disease and do not practicing regular physical exercise. The experimental protocol will begin at least three days after the cardiopulmonary exercise test.  **Fig.1. Experimental Protocol**    During the experimental protocol, all volunteers will perform four experimental sessions, conducted in a random order and with an interval of at least three days between them. Two sessions will be performed in the morning and two in the evening. At each time of day, one control (C) and one exercise (E) sessions will be performed. For each session, volunteers will be asked to arrive to the laborartory using adequate clothes for exercise practice. They will be instructed do not ingest alcohol 24 hours before test, and to avoid performing physical exercises during the 48 hours before the experiments. Windows of the laboratory will stay open during the experimental sessions, temperature will be kept between 22 and 24ºC, and the luminosity will be measured at the beginning and the end of the sessions.  The experimental design of the experimental sessions is showed in Figure 2.  In the morning sessions, the volunteers will arrive to the laboratory at 7 a.m, and in the evening at 5 p.m. For all the sessions, the volunteers will be fasting for at least 5 hours. After arriving, they will receive a standardized meal, composed by two cereal bars and 50ml of juice. Meal will be ingested in 10 to 15 minutes. Then, after emptying their bladder, the volunteers will sit for 60 minutes. Blood sample for noradrenaline, adrenaline, angiotensin II and melatonin dosage will be collected after 45 min of rest. Afterwards, the volunteers will can empty the bladder again and come back to the laboratory, where the experimental devices will be positioned on them. They will stay resting for 15 minutes more to complete the 60 minutes.  At this moment, the pre-intervention period will begin and last 60 minutes. Electrocardiographic, respiratory and BP waves will be recorded for 10 minutes to assess autonomic modulation as previously described. Afterwards, auscultatory BP, HR and CO will be mesured in triplicate. Then, blood flow and vasodilatory capacity of active (leg) and inactive (arm) musculature will be assessed.  After the pre-intervention period, volunteers will be positioned on the cycle ergometer, and will performe the protocol shown in items 4.4.1 for the control sessions or 4.4.2 for the exercise sessions. During the intervention period, eletrocardiographic, respiratory and BP waves will be recorded between 30 and 40 minutes. HR, auscultatory BP and CO will be measured in triplicate between 40 and 50 minutes. VO_2_ will be assessed to verify exercise intensity.  Immediately after the intervention period, the volunteers will come back to the sitting position and the post-intervention period will begin. Auscultatory BP will be assessed every 10 minutes. Electrocardiographic, respiratory and BP waves will be recorded between 10 to 20 minutes and between 35 to 45 minutes. Auscultatory BP, HR and CO will be mesured in triplicate between 20 to 30 minutes and between 50 to 60 minutes. Finally, blood flow and vasodilatory capacity will be assessed between 60 to 80 minutes.  At the end of each session, the devices will be desconected of the volunteers, and they will be asked to take a bath. Afterwards, they will come back to the laboratory and the ambulatory BP monitor will be positioned on their non dominant arm to take measures for the next 24 hours. Volunteers will be instructed to register their daily activities. Moreover, they will be instructed not to drink alcoholic beverages, do exercises, asleep during daytime period and take a bath while using the monitor. Activities and schedule must be the similar after the four experimental sessions.  Fig. 2 – Experimental session    **4.6 Statistical analysis**  Hemodynamic variables (auscultatory BP, CO, HR, SV and SVR) assessed in triplicate at each moment will be analyzed by the mean of the three measure.  Ambulatory BP (AMBP) will be only used if at least 85% of the measured were valid (9). Data will be evaluated in 24 hours, daytime and nightime averages.  Normality of distribution will be tested by Shapiro-Wilks test. Variables that do not present a normal distribution will be mathematically trasnformed.  Hemodynamic and autonomic data will be analyzed by a 3 way ANOVA for repeated measures with main factors: time of day (morning and evening), session (control and exercise) and moment (pre- and post-intervention).  Ambultaory BP will be compared employing a 2 or 3 way ANOVA for repeated measures, establishing as main factors: time of day (morning and evening) and session (control and exercise), and hours when hour averages were analyzed.  Newman Keuls post-hoc test will be employed when necessary. P≤0.05 will be set as significant for all analyzes.  The differences in the behavior between morning and evening will be correlated with the hormonal levels using Pearson or Spearman correlation depending on data distribution.  Considering a power of 80% and an alpha error of 5%, the minimal sample calculated was calculated for different variables: a) to detect a difference of 4 mmHg with standard deviation of 3 mmHg on BP, the minimal sample was calculated to be 9 subjects; b) to detect a difference of 0.32 L/min with a standard deviation of 0.32 L/min on CO, the minimal sample was calculated to be 11 subjects; c) to detect a difference of 0.5 mL.100 mL^-1^ de tissue.min^-1^ with a standard deviation of 0.6 mL.100 mL^-1^ de tissue.min^-1^ on blood flow, the minimal sample was calculated to be 12 subjects. Thus, the proposal sample of 16 subjects will be enough for the 3 main variables of the current study even with some dropout. |
| --- |

**5. Referências**

| 1. III Diretrizes da Sociedade Brasileira de Cardiologia Sobre Teste Ergométrico *Arquivos Brasileiros de Cardiologia* 95: 1-26, 2010.  2. **Aikawa T, Kasahara T, and Uchiyama M.** Circadian variation of plasma arginine vasopressin concentration, or arginine vasopressin in enuresis. *Scand J Urol Nephrol Suppl* 202: 47-49, 1999.  3. **Aires MM.** *Fisiologia*. Rio de Janeiro: Guanabara Koogan, 2008.  4. **Anwar MM, Meki AR, and Rahma HH.** Inhibitory effects of melatonin on vascular reactivity: possible role of vasoactive mediators. *Comp Biochem Physiol C Toxicol Pharmacol* 130: 357-367, 2001.  5. **Araujo EA.** *Respostas Hemodinâmicas e Autonômicas Pós-Exercício: Influência da Massa Muscular, da Intensidade Relativa e do Gasto Energético Total do Exercício*. São Paulo: Universidade de São Paulo, 2007.  6. **Atkinson G, Drust B, Reilly T, and Waterhouse J.** The relevance of melatonin to sports medicine and science. *Sports Med* 33: 809-831, 2003.  7. **Bisquolo VA, Cardoso CG, Jr., Ortega KC, Gusmao JL, Tinucci T, Negrao CE, Wajchenberg BL, Mion D, Jr., and Forjaz CL.** Previous exercise attenuates muscle sympathetic activity and increases blood flow during acute euglycemic hyperinsulinemia. *J Appl Physiol* 98: 866-871, 2005.  8. **Buysse DJ, Reynolds CF, 3rd, Monk TH, Berman SR, and Kupfer DJ.** The Pittsburgh Sleep Quality Index: a new instrument for psychiatric practice and research. *Psychiatry Res* 28: 193-213, 1989.  9. **Cardiologia SBd, Hipertensão SBd, and Nefrologia SBd.** IV Guideline for ambulatory blood pressure monitoring. II Guideline for home blood pressure monitoring. IV ABPM/II HBPM. *Arquivos Brasileiros de Cardiologia* 85: 1-18, 2005.  10. **Cardoso CG, Jr., Gomides RS, Queiroz AC, Pinto LG, da Silveira Lobo F, Tinucci T, Mion D, Jr., and de Moraes Forjaz CL.** Acute and chronic effects of aerobic and resistance exercise on ambulatory blood pressure. *Clinics (Sao Paulo)* 65: 317-325.  11. **Casonato J PM, D.** Hipotensão pós-exercício aeróbico: uma revisão sistemática. *Revista Brsileira de Medicina do Esporte* 15: 151-157, 2009.  12. **Chobanian AV, Bakris GL, Black HR, Cushman WC, Green LA, Izzo JL, Jr., Jones DW, Materson BJ, Oparil S, Wright JT, Jr., and Roccella EJ.** Seventh report of the Joint National Committee on Prevention, Detection, Evaluation, and Treatment of High Blood Pressure. *Hypertension* 42: 1206-1252, 2003.  13. **Cleroux J, Kouame N, Nadeau A, Coulombe D, and Lacourciere Y.** Aftereffects of exercise on regional and systemic hemodynamics in hypertension. *Hypertension* 19: 183-191, 1992.  14. **Collier CR.** Determination of mixed venous CO2 tensions by rebreathing. *J Appl Physiol* 9: 25-29, 1956.  15. **Cugini P and Lucia P.** [Circadian rhythm of the renin-angiotensin-aldosterone system: a summary of our research studies]. *Clin Ter* 155: 287-291, 2004.  16. **Davies GJ, Chierchia S, and Maseri A.** Prevention of myocardial infarction by very early treatment with intracoronary streptokinase. Some clinical observations. *N Engl J Med* 311: 1488-1492, 1984.  17. **Dimitrov S, Benedict C, Heutling D, Westermann J, Born J, and Lange T.** Cortisol and epinephrine control opposing circadian rhythms in T cell subsets. *Blood* 113: 5134-5143, 2009.  18. **Dujic Z, Ivancev V, Valic Z, Bakovic D, Marinovic-Terzic I, Eterovic D, and Wisloff U.** Postexercise hypotension in moderately trained athletes after maximal exercise. *Med Sci Sports Exerc* 38: 318-322, 2006.  19. **Fagard RH.** Effects of exercise, diet and their combination on blood pressure. *J Hum Hypertens* 19 Suppl 3: S20-24, 2005.  20. **Forjaz CL, Cardoso CG, Jr., Rezk CC, Santaella DF, and Tinucci T.** Postexercise hypotension and hemodynamics: the role of exercise intensity. *J Sports Med Phys Fitness* 44: 54-62, 2004.  21. **Forjaz CL, Matsudaira Y, Rodrigues FB, Nunes N, and Negrao CE.** Post-exercise changes in blood pressure, heart rate and rate pressure product at different exercise intensities in normotensive humans. *Braz J Med Biol Res* 31: 1247-1255, 1998.  22. **Forjaz CL, Ramires PR, Tinucci T, Ortega KC, Salomao HE, Ignes EC, Wajchenberg BL, Negrao CE, and Mion D, Jr.** Postexercise responses of muscle sympathetic nerve activity and blood flow to hyperinsulinemia in humans. *J Appl Physiol* 87: 824-829, 1999.  23. **Forjaz CL, Santaella DF, Rezende LO, Barretto AC, and Negrao CE.** [Effect of exercise duration on the magnitude and duration of post-exercise hypotension]. *Arq Bras Cardiol* 70: 99-104, 1998.  24. **Forjaz CL, Tinucci T, Ortega KC, Santaella DF, Mion D, Jr., and Negrao CE.** Factors affecting post-exercise hypotension in normotensive and hypertensive humans. *Blood Press Monit* 5: 255-262, 2000.  25. **Grassi G, Bombelli M, Seravalle G, Dell'Oro R, and Quarti-Trevano F.** Diurnal blood pressure variation and sympathetic activity. *Hypertens Res* 33: 381-385.  26. **Hagberg JM, Montain SJ, and Martin WH, 3rd.** Blood pressure and hemodynamic responses after exercise in older hypertensives. *J Appl Physiol* 63: 270-276, 1987.  27. **Halliwill JR.** Mechanisms and clinical implications of post-exercise hypotension in humans. *Exerc Sport Sci Rev* 29: 65-70, 2001.  28. **Halliwill JR, Taylor JA, and Eckberg DL.** Impaired sympathetic vascular regulation in humans after acute dynamic exercise. *J Physiol* 495 ( Pt 1): 279-288, 1996.  29. **Halliwill JR, Taylor JA, Hartwig TD, and Eckberg DL.** Augmented baroreflex heart rate gain after moderate-intensity, dynamic exercise. *Am J Physiol* 270: R420-426, 1996.  30. **Hamer M and Boutcher SH.** Impact of moderate overweight and body composition on postexercise hemodynamic responses in healthy men. *J Hum Hypertens* 20: 612-617, 2006.  31. **Harvey PJ, Morris BL, Kubo T, Picton PE, Su WS, Notarius CF, and Floras JS.** Hemodynamic after-effects of acute dynamic exercise in sedentary normotensive postmenopausal women. *J Hypertens* 23: 285-292, 2005.  32. **Jones H, George K, Edwards B, and Atkinson G.** Effects of time of day on post-exercise blood pressure: circadian or sleep-related influences? *Chronobiol Int* 25: 987-998, 2008.  33. **Jones H, George K, Edwards B, and Atkinson G.** Is the magnitude of acute post-exercise hypotension mediated by exercise intensity or total work done? *Eur J Appl Physiol* 102: 33-40, 2007.  34. **Jones H, Green DJ, George K, and Atkinson G.** Intermittent exercise abolishes the diurnal variation in endothelial-dependent flow-mediated dilation in humans. *Am J Physiol Regul Integr Comp Physiol* 298: R427-432, 2010.  35. **Jones H, Pritchard C, George K, Edwards B, and Atkinson G.** The acute post-exercise response of blood pressure varies with time of day. *Eur J Appl Physiol* 104: 481-489, 2008.  36. **Jones H, Taylor CE, Lewis NC, George K, and Atkinson G.** Post-exercise blood pressure reduction is greater following intermittent than continuous exercise and is influenced less by diurnal variation. *Chronobiol Int* 26: 293-306, 2009.  37. **Kawano H, Motoyama T, Yasue H, Hirai N, Waly HM, Kugiyama K, and Ogawa H.** Endothelial function fluctuates with diurnal variation in the frequency of ischemic episodes in patients with variant angina. *J Am Coll Cardiol* 40: 266-270, 2002.  38. **Kenney MJ and Seals DR.** Postexercise hypotension. Key features, mechanisms, and clinical significance. *Hypertension* 22: 653-664, 1993.  39. **Kitajima T, Kanbayashi T, Saitoh Y, Ogawa Y, Sugiyama T, Kaneko Y, Sasaki Y, Aizawa R, and Shimisu T.** The effects of oral melatonin on the autonomic function in healthy subjects. *Psychiatry Clin Neurosci* 55: 299-300, 2001.  40. **Legramante JM, Galante A, Massaro M, Attanasio A, Raimondi G, Pigozzi F, and Iellamo F.** Hemodynamic and autonomic correlates of postexercise hypotension in patients with mild hypertension. *Am J Physiol Regul Integr Comp Physiol* 282: R1037-1043, 2002.  41. **Lewis NC, Atkinson G, Lucas SJ, Grant EJ, Jones H, Tzeng YC, Horsman H, and Ainslie PN.** Diurnal variation in time to presyncope and associated circulatory changes during a controlled orthostatic challenge. *Am J Physiol Regul Integr Comp Physiol* 299: R55-61, 2010.  42. **Lockwood JM, Pricher MP, Wilkins BW, Holowatz LA, and Halliwill JR.** Postexercise hypotension is not explained by a prostaglandin-dependent peripheral vasodilation. *J Appl Physiol* 98: 447-453, 2005.  43. **Lockwood JM, Wilkins BW, and Halliwill JR.** H1 receptor-mediated vasodilatation contributes to postexercise hypotension. *J Physiol* 563: 633-642, 2005.  44. **Lombardi F, Sandrone G, Mortara A, La Rovere MT, Colombo E, Guzzetti S, and Malliani A.** Circadian variation of spectral indices of heart rate variability after myocardial infarction. *Am Heart J* 123: 1521-1529, 1992.  45. **Lynn BM, Minson CT, and Halliwill JR.** Fluid replacement and heat stress during exercise alter post-exercise cardiac haemodynamics in endurance exercise-trained men. *J Physiol* 587: 3605-3617, 2009.  46. **M DEZ, Covassin N, G DEMT, Sarlo M, and Stegagno L.** Sleep onset and cardiovascular activity in primary insomnia. *J Sleep Res*.  47. **MacDonald JR.** Potential causes, mechanisms, and implications of post exercise hypotension. *J Hum Hypertens* 16: 225-236, 2002.  48. **McCord JL, Beasley JM, and Halliwill JR.** H2-receptor-mediated vasodilation contributes to postexercise hypotension. *J Appl Physiol* 100: 67-75, 2006.  49. **Millar-Craig MW, Bishop CN, and Raftery EB.** Circadian variation of blood-pressure. *Lancet* 1: 795-797, 1978.  50. **Nakagawa H and Okumura N.** Coordinated regulation of circadian rhythms and homeostasis by the suprachiasmatic nucleus. *Proc Jpn Acad Ser B Phys Biol Sci* 86: 391-409.  51. **NIH. National Institute of Health.** The Practical Guide Identification, Evaluation, and Treatment of Overweight and Obesity in Adults. *NIH Publicatio*: 1-75, 2000.  52. **Notarius CF, Morris BL, and Floras JS.** Caffeine attenuates early post-exercise hypotension in middle-aged subjects. *Am J Hypertens* 19: 184-188, 2006.  53. **Otto ME, Svatikova A, Barretto RB, Santos S, Hoffmann M, Khandheria B, and Somers V.** Early morning attenuation of endothelial function in healthy humans. *Circulation* 109: 2507-2510, 2004.  54. **Pagani M, Somers V, Furlan R, Dell'Orto S, Conway J, Baselli G, Cerutti S, Sleight P, and Malliani A.** Changes in autonomic regulation induced by physical training in mild hypertension. *Hypertension* 12: 600-610, 1988.  55. **Panza JA, Epstein SE, and Quyyumi AA.** Circadian variation in vascular tone and its relation to alpha-sympathetic vasoconstrictor activity. *N Engl J Med* 325: 986-990, 1991.  56. **Park SW, Choi SM, and Lee SM.** Effect of melatonin on altered expression of vasoregulatory genes during hepatic ischemia/reperfusion. *Arch Pharm Res* 30: 1619-1624, 2007.  57. **Pescatello LS, Franklin BA, Fagard R, Farquhar WB, Kelley GA, and Ray CA.** American College of Sports Medicine position stand. Exercise and hypertension. *Med Sci Sports Exerc* 36: 533-553, 2004.  58. **Rondon MUPB, Alves MJNN, Braga AMFW, Teixeira OTUN, Barreto ACP, Krieger EM, and Negrão CE.** Postexercise Blood Pressure Reduction in Elderly Hypertensive Patients. *Journal of the American College of Cardiology* 39: 676-682, 2002.  59. **Sociedade Brasileira de Hipertensão.** VI Diretrizes Brasileiras de Hipertensão. *Revista Hipertensão* 13: 1-68, 2010.  60. **Scheer FA, Kalsbeek A, and Buijs RM.** Cardiovascular control by the suprachiasmatic nucleus: neural and neuroendocrine mechanisms in human and rat. *Biol Chem* 384: 697-709, 2003.  61. **Scott JM, Esch BT, Lusina SJ, McKenzie DC, Koehle MS, Sheel AW, and Warburton DE.** Post-exercise hypotension and cardiovascular responses to moderate orthostatic stress in endurance-trained males. *Appl Physiol Nutr Metab* 33: 246-253, 2008.  62. **Senitko AN, Charkoudian N, and Halliwill JR.** Influence of endurance exercise training status and gender on postexercise hypotension. *J Appl Physiol* 92: 2368-2374, 2002.  63. **Task Force of the European Society of Cardiology and the North American Society of Pacing and Electrophysiology.** Heart rate variability: standards of measurement, physiological interpretation and clinical use. Task Force of the European Society of Cardiology and the North American Society of Pacing and Electrophysiology. *Circulation* 93: 1043-1065, 1996.  64. **Teixeira L.** *Efeito isolado e associado do exercício aeróbio e resistido na pressão arterial pós-exercício e seus mecanismos hemodinâmicos, neurais e de estado de ansiedade* (Mestrado). São Paulo: Universidade de São Paulo, 2007.  65. **Tochikubo O, Kawano Y, Miyajima E, Toshihiro N, and Ishii M.** Circadian variation of hemodynamics and baroreflex functions in patients with essential hypertension. *Hypertens Res* 20: 157-166, 1997.  66. **Turner MJ, Tanaka H, Bassett DR, Jr., and Fitton TR.** The equilibrium CO2 rebreathing method does not affect resting or exercise blood pressure. *Med Sci Sports Exerc* 28: 921-925, 1996.  67. **Van de Borne P, Nguyen H, Biston P, Linkowski P, and Degaute JP.** Effects of wake and sleep stages on the 24-h autonomic control of blood pressure and heart rate in recumbent men. *Am J Physiol* 266: H548-554, 1994.  68. **Vanhees L, Defoor J, Schepers D, Brusselle S, Reybrouck T, and Fagard R.** Comparison of cardiac output measured by two automated methods of CO2 rebreathing. *Med Sci Sports Exerc* 32: 1028-1034, 2000.  69. **Vogel RA.** Measurement of endothelial function by brachial artery flow-mediated vasodilation. *Am J Cardiol* 88: 31E-34E, 2001.  70. **Voogel AJ, Koopman MG, Hart AA, van Montfrans GA, and Arisz L.** Circadian rhythms in systemic hemodynamics and renal function in healthy subjects and patients with nephrotic syndrome. *Kidney Int* 59: 1873-1880, 2001.  71. **Wilkinson IB and Webb DJ.** Venous occlusion plethysmography in cardiovascular research: methodology and clinical applications. *Br J Clin Pharmacol* 52: 631-646, 2001. |
| --- |
