# Supplementary material for: Post-Exercise Hypotension and Its Mechanisms Differ after Morning and Evening Exercise: A Randomized Crossover Study
Source: PLoS One. 2015 Jul 17;10(7):e0132458. doi: 10.1371/journal.pone.0132458 (PMC4506120; doi:10.1371/journal.pone.0132458)
Supplement: S3 File — (PDF) [file pone.0132458.s004.pdf]

Proof-Reading-Service.com
PhD theses, journal papers, books and other professional documents

Proof-Reading-Service.com Ltd, Devonshire Business Centre, Works Road, Letchworth Garden City, Hertfordshire, SG6 1GJ, United Kingdom
Office phone: +44(0)20 31 500 431
E-mail: enquiries@proof-reading-service.com
Internet: http://www.proof-reading-service.com
VAT registration number: 911 4788 21
Company registration number: 8391405

Receipt Address
Leandro Brito
Universidade de São Paulo
São Paulo, 05508-000
Brazil

DATE
19 May 2015

INVOICE #
201505-20526

DESCRIPTION	WORD COUNT	PRICE per 1,000 words	TOTAL
Leandro_Brito_19052015	4827	£8.99	**£43.39**
		Subtotal	**£43.39**
		Credit Card Charges	**£4.33**
		VAT @ 20.00%	**£9.55**
		**Total**	**£57.27**

The above amount has been paid through PayPal.

THANK YOU FOR YOUR BUSINESS
